# Osteopontin—A Potential Biomarker for IgA Nephropathy: Machine Learning Application

**DOI:** 10.3390/biomedicines10040734

**Published:** 2022-03-22

**Authors:** Barbara Moszczuk, Natalia Krata, Witold Rudnicki, Bartosz Foroncewicz, Dominik Cysewski, Leszek Pączek, Beata Kaleta, Krzysztof Mucha

**Affiliations:** 1 Department of Immunology, Transplantology and Internal Diseases, Medical University of Warsaw, 02-006 Warsaw, Poland; barbara.moszczuk@wum.edu.pl (B.M.); nkrata@wum.edu.pl (N.K.); bartosz.foroncewicz@wum.edu.pl (B.F.); leszek.paczek@wum.edu.pl (L.P.); 2 ProMix Center (ProteogenOmix in Medicine), Department of Immunology, Transplantology and Internal Diseases, Medical University of Warsaw, 02-006 Warsaw, Poland; 3 Department of Clinical Immunology, Medical University of Warsaw, 02-006 Warsaw, Poland; 4 Computational Centre and Institute of Computer Science, University of Białystok, 15-245 Białystok, Poland; w.rudnicki@uwb.edu.pl; 5 Interdisciplinary Centre for Mathematical and Computational Modelling, University of Warsaw, 02-630 Warsaw, Poland; 6 Institute of Biochemistry and Biophysics, Polish Academy of Sciences, 02-106 Warsaw, Poland; dominikcysewski@gmail.com

**Keywords:** biomarkers, IgA nephropathy, lupus nephritis, machine learning, membranous nephropathy, osteopontin, peroxiredoxins

## Abstract

Many potential biomarkers in nephrology have been studied, but few are currently used in clinical practice. One is osteopontin (OPN). We compared urinary OPN concentrations in 80 participants: 67 patients with various biopsy-proven glomerulopathies (GNs)—immunoglobulin A nephropathy (IgAN, 29), membranous nephropathy (MN, 20) and lupus nephritis (LN, 18) and 13 with no GN. Follow-up included 48 participants. Machine learning was used to correlate OPN with other factors to classify patients by GN type. The resulting algorithm had an accuracy of 87% in differentiating IgAN from other GNs using urinary OPN levels only. A lesser effect for discriminating MN and LN was observed. However, the lower number of patients and the phenotypic heterogeneity of MN and LN might have affected those results. OPN was significantly higher in IgAN at baseline than in other GNs and therefore might be useful for identifying patients with IgAN. That observation did not apply to either patients with IgAN at follow-up or to patients with other GNs. OPN seems to be a valuable biomarker and should be validated in future studies. Machine learning is a powerful tool that, compared with traditional statistical methods, can be also applied to smaller datasets.

## 1. Introduction

According to the U.S. Centers for Disease Control and Prevention, the number of people affected with chronic kidney disease (CKD) in the United States has reached 37 million—15% of the adult population [1]. In 2018, the leading causes of end-stage kidney disease were diabetes (39%), hypertension (26%), and glomerulonephritis (15%). Those conditions can present a similar clinical picture or can overlap, necessitating the use of invasive diagnostic methods such as kidney biopsy. The need to define and implement noninvasive diagnostic markers is particularly pressing in the immune-related glomerulonephropathies (GNs), whose treatment is different from that for diabetes- or hypertension-related CKD. Efforts to create noninvasive tests that will help diagnose and monitor kidney disease have included genomic, transcriptomic, and proteomic approaches to detect gene polymorphisms [2,3], mRNA expression [4], and serum and urinary proteins [5,6]. Unfortunately, new biomarkers are not used in everyday clinical practice, mostly because of insufficient diagnostic sensitivity and specificity as demonstrated in clinical trials. Thus, the search for clinically useful biomarkers in CKD continues.

Osteopontin (OPN) is a multifunctional, extracellular phosphoprotein that is expressed in various cells and tissues, including fibroblasts, osteoblasts, macrophages, endothelial cells, adipocytes, Kupffer cells, and dendritic cells. Studies have demonstrated that OPN plays a role in the development of inflammation, wound healing, cancer metastases, diabetes, nephrolithiasis, and modulation of osteoclast function (reviewed in [7]).

The role of OPN in glomerular diseases is not clearly defined. OPN gene polymorphisms are associated with the development of diabetic nephropathy in type 2 diabetes [8], urinary OPN (uOPN) excretion in patients with IgA nephropathy (IgAN) [9], and acute renal allograft rejection [10]. OPN mRNA expression in tissue is increased in areas of tubular damage [11] and in patients with renal calculi [12]. Interestingly, serum OPN has been confirmed to be a biomarker correlating with renal involvement in patients with systemic lupus erythematosus [13] and to be independently associated with the development of microalbuminuria in patients with type 1 diabetes mellitus [14]. Finally, urinary OPN is known to rise in active lupus nephritis (LN) [15]. However, OPN as a factor for discriminating between various kidney diseases has not yet been fully explored. In previous research, our team focused on the link between OPN gene polymorphisms and excretion of uOPN in patients with IgAN [9]. In the present study, we compared uOPN in various immune-related glomerulopathies. IgAN is the most common primary GN, with an incidence of 2–5 adults per 100,000 [16]. Primary membranous nephropathy (MN) is a rare disease (ORPHA number 97,560), but an important cause of proteinuria. LN is a frequent secondary autoimmune GN with variable histopathologic picture. We measured uOPN concentrations in patients with those GNs to assess the potential utility of uOPN as a biomarker. We also compared uOPN with concentrations of peroxiredoxins (PRDXs), previously studied markers of oxidative stress [5], creating a network of biologic pathways that involve OPN to better understand the role of OPN in cells.

Our aim in the present study was to compare uOPN concentrations in patients with various GNs and to use machine learning (ML) to correlate those concentrations with clinical factors and with PRDXs.

## 2. Materials and Methods

### 2.1. Patients and Healthy Participants

OPN at baseline was measured in 80 participants: 67 patients—IgAN (*n* = 29), LN (*n* = 18), MN (*n* = 20) and 13 healthy individuals defined by the absence of any kidney disease or other chronic diseases requiring treatment. Measurement from 48 participants were available during follow-up: 43 patients—IgAN (*n* = 18), LN (*n* = 11), MN (*n* = 14) and 5 healthy individuals. IgAN, LN, and MN were confirmed by renal biopsy. The healthy control group consisted of age- and sex-matched volunteers. Exclusion criteria were active infection, current pregnancy, history of malignancy, or prior organ transplantation. Written informed consent was given by all study participants. Table 1 and Table 2 present the clinical characteristics of the study participants.

We measured uOPN in the participants at two separate time points: baseline and follow-up. The study period for IgAN and LN was October 2015–October 2018, MN: October 2016–October 2018, Control: April 2017–November 2018. The average and standard deviation of the follow-up period was 27.79 ± 7.85 months for OPN and 45.56 ± 18.45 months for estimated glomerular filtration rate (eGFR), body mass index, and 24 h proteinuria.

The study was approved by the Ethics Committee of the Medical University of Warsaw, nos.: KB/9/2010 (26 January 2010) and KB/199/2016 (11 October 2016). All study procedures proceeded in accordance with the Helsinki Declaration of 1975, revised in 2000.

### 2.2. Methods

#### 2.2.1. Material Collection

Urine samples (second or third morning urine) were centrifuged (10 min at 2000 rpm) within 120 min from collection, aliquoted into 2 mL cryovials, and frozen at −80 °C until use. Laboratory tests such as serum creatinine, blood morphology, urinalysis, and urinary protein were performed using routine laboratory techniques. PRDX concentrations had been obtained during a previous study of the same patient sample [5]. The eGFR was calculated using the chronic kidney disease epidemiology collaboration (CKD-EPI) equation. Body weight in kilograms was divided by the square of the height in meters (kg/m^2^) to evaluate body mass index.

#### 2.2.2. OPN Measurements

OPN was measured with the Human Osteopontin (OPN) Quantikine ELISA Kit (R&D Systems, Minneapolis, MN, USA). Urine samples were diluted 20× with assay diluent according to the manufacturer’s instructions. To each well in the 96-well microplate (precoated with a monoclonal antibody specific for human OPN), 100 μL of assay diluent was added; then, 50 μL each of standard and sample were pipetted into the wells in duplicate. The microplate was then incubated for 2 h at room temperature (22–25 °C), allowing the OPN in the sample to be bound by the immobilized antibody. After incubation, any unbound substances were washed away manually using a wash buffer provided by the manufacturer and according to the assay procedure, and 200 μL of an enzyme-linked polyclonal antibody specific for human OPN was added to the wells. The plate was again incubated for 2 h at room temperature (22–25 °C). After a wash to remove any unbound antibody–enzyme reagent, 200 μL of a substrate solution was added to the wells, where color developed in proportion to the amount of OPN bound in the initial step. The color development was stopped by the addition of the stop solution included with the assay, and the optical density was subsequently measured using a BioTek PowerWave XS microplate reader (Agilent, Santa Clara, CA, USA) at a wavelength of 450 nm. To determine the OPN concentration (ng/mL), the GraphPad Prism software application (version 9.0.1: GraphPad Software, San Diego, CA, USA) used the optical density with a standard curve (4-parameter logistic equation), including extrapolation. Each result was multiplied by 20 to obtain the actual urine OPN concentration.

### 2.3. Statistical Analysis

#### 2.3.1. Demographic Data and OPN Measurements

The statistical analysis was performed in the GraphPad Prism (version 9.0.1) and Statistica (version 13.1, StatSoft, Tulsa, OK, USA) software applications. Results are expressed as mean ± standard deviation, median ± interquartile range, or a percentage. All variables were examined by the Shapiro–Wilk test for normal distribution. Non-normally distributed variables were analyzed using nonparametric tests. Comparisons between demographic variables were tested using the Kruskal–Wallis test and between the control and GN groups, using the Mann–Whitney *U*-test. Correlations between pairs of parameters were examined using Spearman’s correlation analysis., The differences between categorical variables were calculated with Chi square test. The level of significance was set to *p* < 0.05.

#### 2.3.2. Implementation of ML and Mathematical Analysis

We performed analysis of the data set using an approach based on the supervised machine learning algorithms. Application of machine learning (ML) allows us to perform rapid exploration of data without prior statements of detailed models and with minimal assumptions about data. ML also automatically includes interactions between variables into account. All findings of the ML approach were verified with the help of statistical analysis, and very good agreement between both methods was obtained, in particular when a strong signal was obtained. We used the ML algorithm Random Forest [17] to build a model that used standard clinical indicators, together with OPN and PRDX levels to predict each participant’s classification: control, IgAN, MN, and LN. Given the available data collected, eight descriptors were available for all 80 patients: “Gndr” (gender), “BMI” (body mass index), “CR” (creatinine), “eGFR,” ”Hb” (hemoglobin), “PLT” (platelets), ”WBC” (white blood cells), and “OPN”. PRDX levels (1–5) were available for only 53 patients: 7 in the control class, 16 in the IgAN class, 12 in the LN class, and 16 in the MN class.

The analysis consisted of two steps. In the first step, the all-relevant-features selection algorithm Boruta [18] was used to find the descriptive variables carried information about the class variable. Then the Random Forest algorithm was built using only the variables not rejected by Boruta. We used the Random Forest [19] and Boruta [20] libraries in R [21]. Random Forest is a general-purpose ML algorithm for classification and nonparametric regression, widely used across multiple disciplines. It is an ensemble of decision trees. Each tree is built using a different data sample, and each split in a tree is built on a variable selected from a subset of all variables. A subset of the objects not used for the construction of a particular tree—the so-called out-of-bag objects—can be used for an unbiased estimate of the classification error and variable importance. In particular, the importance of a variable is established by measuring the decrease in the accuracy with which out-of-bag objects are classified when information about the variable under consideration is removed from the trees.

The Boruta algorithm belongs to the class of all-relevant-features selection algorithms. It is a wrapper around Random Forest. It works by extending the original set of variables by their randomized copies, so-called shadow variables. By design, the randomized copies carry no information about the decision variable. Boruta builds multiple Random Forest classifiers, each using a different set of shadow variables, and compares the importance of the original variables with the importance of the most important shadow variable (shadow max) from each set of shadow variables. The variables whose importance exceeds that of the most important shadow variable in a statistically significant way are deemed relevant. The variables that are statistically less important than the most important shadow variable are deemed irrelevant. The variables for which the test is inconclusive are called tentative. A full description of applied algorithms is available in Supplement, File S1.

Due to the randomized character of used algorithms, the results may minimally differ between each calculation.

## 3. Results

Our results are divided into three sections. First is the analysis performed with ML. An algorithm was introduced to a set of laboratory data and biopsy-proven diagnoses from half the samples, thus “teaching” the algorithm to form diagnostic pathways (decision trees based on yes/no commands). Those decision trees were then applied to a set of data without a known diagnosis to test their accuracy (Section 3.1, Section 3.2 and Section 3.3). Second is a comparison of OPN levels at the approximate time of diagnosis and after treatment in all tested groups, which checked for correlations with clinical factors (Section 3.4). Third is the creation, using the information previously obtained, of a network of biologic processes that includes OPN.

### 3.1. Whole-Group Analysis

**Figure 1:**
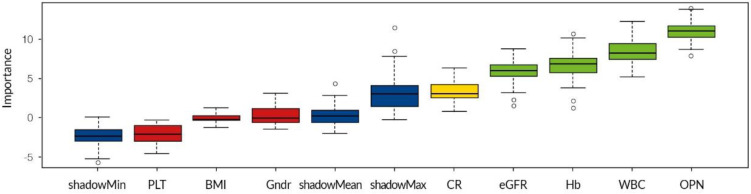
Variables designated as important in the whole-group analysis: green = strong correlation; yellow = marginal correlation; CR = mean serum creatinine; Hb = mean hemoglobin concentration; WBC = mean white blood cell count; PLT = mean platelet concentration; Gndr = gender; BMI = body mass index; eGFR = mean estimated glomerular filtration rate calculated using the chronic kidney disease epidemiology collaboration equation; OPN = osteopontin (first sampling point). Mean values of selected parameters are the average of all measurements of each parameter during long-term follow-up for each patient.

Based on the provided data, the algorithm “decides” which sample matches which GN. However, not every variable has equal significance. Figure 1 shows the variables that were selected by the algorithm as important in correctly placing a patient into a given GN class. Data are tested against the shadow values created by the Boruta algorithm to establish a variable’s significance.

We compared the importance of the variables marked as significant by the algorithm in correctly classifying a patient to a GN class (Table 3). A higher value indicates a higher error in patient placement when the variable is removed from the dataset. Each entry corresponds to an average decrease in the accuracy of decision trees for objects of a particular class when information concerning a given variable is withdrawn from the classifier. The last column is the average value regardless of class. Rows are sorted in descending order based on mean importance. OPN is most responsible for correct patient placement in the IgAN class. Mean eGFR is the most important variable for correct classification of healthy participants, and Hb and WBC are the most important for LN. The quality of prediction is worst for the MN class, with no variable being relevant for that class. Table 4 shows the accuracy of the algorithm based on results from 10 runs of the classifier. The algorithm was not able to correctly classify the control participants (probably because too few samples were available), but the prediction was correct most of the time for patients with IgAN (class error 0.31).

### 3.2. Analysis for IgAN Compared with Other Groups

Using the previously selected variables (OPN, WBC, Hb, eGFR, and CR), we tested our algorithm by finding patients with IgAN from among other non-IgAN samples. In Table 5, a strong confirmation of the relevance of OPN for correctly placing a patient into the IgAN class is evident (the highest number in the column). Each entry corresponds to an average decrease in the accuracy of decision trees for objects of a particular class when information about a given variable is withdrawn from the classifier. The last column is average value regardless of class. Rows appear in descending order based on mean importance. Four other variables are relevant as well.

**Figure 2:**
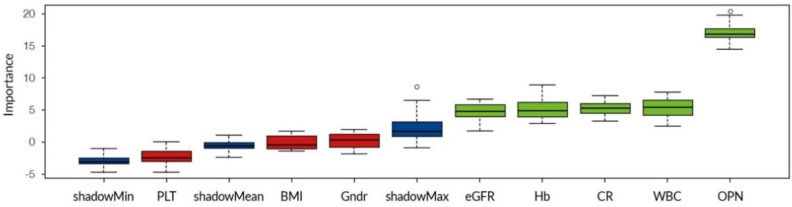
Relevance of variables in correctly enrolling a sample to an IgAN class created by the Boruta algorithm.

Ten runs of the Random Forest classifier were performed. The results are nonrandom, as shown in Table 6. OPN had the strongest prediction value for IgAN, with a low error margin: class error 0.13, which means that the algorithm was 87% correct. OPN had no predictive value for LN or MN. (Hb and WBC did but are not shown. Data available on request). Figure 2 shows the results in pictorial form.

### 3.3. Analysis for OPN Compared with PRDX

In previous research, our team studied PRDXs as potential biomarkers of oxidative stress in IgAN, MN, and LN [5]. We observed that the concentration of PRDXs 1–5 differed in patients with various GNs. For the present study, we added PRDXs to a whole-group analysis similar to the one described in Section 3.2. The previously used variables and PRDXs 1–5 were tested for their prediction strength in placing a patient into the correct class (Table 7). Only two PRDXs are shown because the others failed to achieve the required accuracy (importance was measured on the reduced dataset consisting of the 53 patients for whom the additional measurements of PRDX protein levels were available).

**Figure 3:**
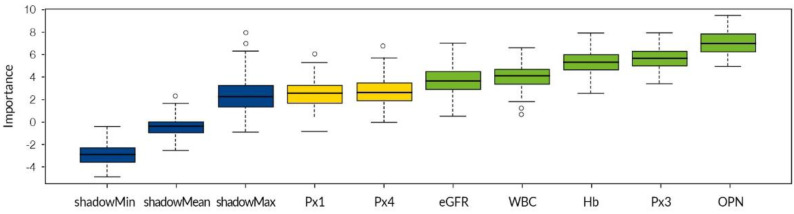
Data tested against “shadow values” created by the Boruta algorithm. Peroxiredoxin (Px) 3 performs best but is weaker than osteopontin (OPN): eGFR = estimated glomerular filtration rate; WBC = white blood cells; Hb = hemoglobin.

OPN was again the key variable for correctly enrolling patients into the IgAN class. Mean eGFR was the most important variable for healthy participants, and WBC and Hb were important for predicting the LN class. However, PRDX3 was now equally as strong as WBC for the LN class, and it also appears to be relevant for the MN class (together with eGFR in the latter case). In contrast to the results presented in Table 3, two variables are now relevant for predicting the MN class. Interestingly, PRDX4 remained classified as “tentative” (uncertain) by Boruta and it seems to contribute some information to LN class prediction. Furthermore, the classification error for MN prediction improved significantly, as shown in Table 8. On the other hand, the predictions for healthy participants are now wrong. Figure 3 shows those results in pictorial form.

### 3.4. Comparison of Various GNs: Standard Modelling

**Figure 4:**
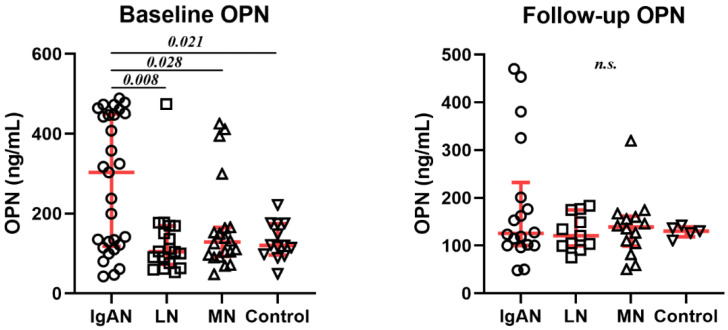
Levels of osteopontin (OPN) in 80 patients at baseline and 48 patients at follow-up are significantly different in the immunoglobulin A nephropathy (IgAN) class. Values are presented as a scatter-dot plot with median (middle line), lower (25%), and upper (75%) quartile (as whiskers). The *p*-value was calculated with the nonparametric Mann–Whitney *U* Test; LN = lupus nephritis; MN = membranous nephropathy, n.s.—not significant.

As mentioned in Section 2.2, OPN was measured at two time points: shortly after diagnosis and after a mean follow-up of 27.8 months. Table 1 presents that 100% of GN patients received ACEi or angiotensin receptor blocker at both time points. There was a difference in immunosuppression treatment that was received by 71–75% of MN, 91–94% of LN, and 28–34% of IgAN patients. Figure 4 shows the values at both time points. OPN is clearly no longer a differentiating factor.

The Spearmann correlation analysis summarized in Table 9 shows some level of association between OPN in patients with IgAN at follow-up and with PLTs. (The remaining correlations are available in Supplement, Table S1).

### 3.5. OPN in the Setting of the Cell Proteome

**Figure 5:**
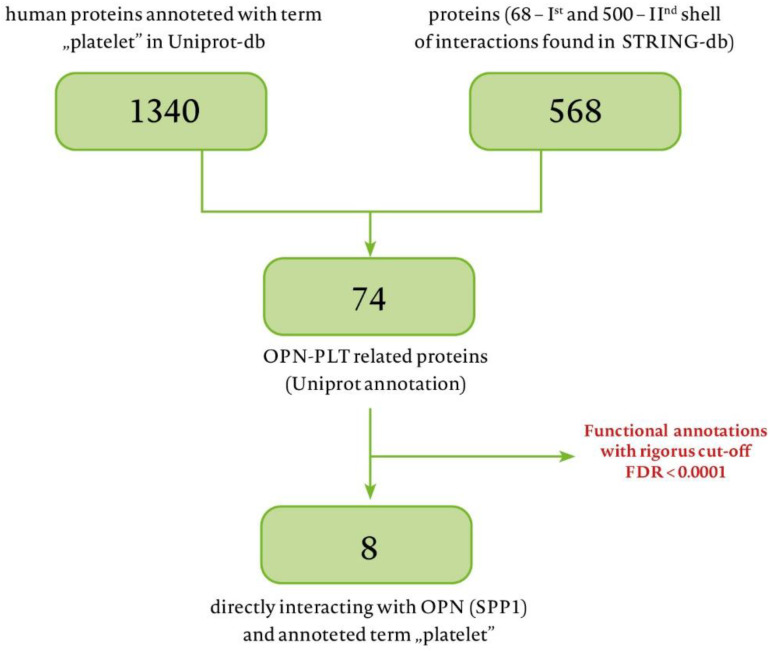
Selection of proteins that interact with osteopontin (OPN) from the STRING-db database: FDR = false discovery rate.

To investigate the potential relationship between the OPN and platelets [22,23], we searched the Uniport database for all human proteins annotated with the term “platelets” (PLT) receiving 1340 proteins. Using the STRING-database, we selected proteins that interacted directly or indirectly with OPN. We adopted an increased value of data reliability (term: “high confidence”). This way, we received 68 direct-interacting proteins and 500 that make up the second layer. A total of 500 proteins of the second interaction layer are the maximum number of proteins that can be indicated by this algorithm. A comparison of these two lists results in 74 proteins annotated with the term “platelets” and interact with OPN directly or via maximum one mediating protein (Supplement, File S2). Out of 74 proteins, eight are direct-interacting OPN-PLT proteins (process shown in Figure 5). To interpret the results more broadly, we performed functional enrichment. We set the cut-off point at FDR > 0.0001, or at the limit of the top 100 results for a given category [24,25].

We also prepared a functional analysis of selected proteins to evaluate their role in various biologic pathways, with eight proteins being functionally analyzed in gene ontology terms (an adjusted *p* value < 0.0001 was considered significant). The Kyoto Encyclopedia of Genes and Genomes was used to select the major biologic pathway–based target gene [26]. A path comprises a minimum of two genes. The *p* value obtained from each biologic route was adjusted using the Benjamini–Hochberg false discovery rate procedure [27]. Biologic pathways with a false discovery rate less than 0.0001 were considered significant. Table 10 summarizes the selected pathways and processes.

## 4. Discussion

In our opinion, a major finding of this study is that the identification of biomarkers in nephrology might be empowered by ML. ML has recently become widely used in numerous biomedical applications ranging from the analysis of Parkinson’s disease [28], through the prediction of COVID-19 patient health [29], to spectacularly accurate predictions of three-dimensional protein structure [30]. ML methods complement the traditional statistical analysis for problems that involve complex relationships between various parameters of the studied phenomena and allow us to obtain predictive models in such situations. In particular, ML methods are widely used for identification of biomarkers [31] for diseases with complex and not-well understood mechanisms. The general idea is straightforward—if a robust predictive model can be obtained for the process under scrutiny, the variables that are used by the model are necessarily connected to this process, even if we currently do not understand why and how they are connected. Such variables can be then used as biomarkers. Moreover, they also can foster understanding by focusing experimental effort.

Random Forest [31] was used in the present work as both a classifier and an engine for the feature selection study goals. In the thorough review of 179 classifiers from 17 families, performed on 121 data sets, classifiers from the Random Forest family have shown the best and the most robust performance [31,32]. The all-relevant feature selection algorithm Boruta [31], a wrapper using Random Forest, was used for identification of relevant biomarkers. It was tested on a wide range of problems, and several recently published real-world datasets showed that the algorithm is both sensitive and selective [33].

In nephrology, ML has been applied to the prediction of IgAN progression to end-stage kidney disease, identification of diabetic and nondiabetic renal disorders, assessment of acute kidney injury, and dialysis-associated death [34]. In the present study, we used ML algorithms to select relevant variables from a clinical dataset, and we then applied them to distinguish various GN classes. Even applied to small groups, the algorithm correctly identified patients with IgAN, as confirmed by biopsy and a standard Mann–Whitney *U*-test. Interestingly, OPN was observed to be more specific than PRDX in the selected subgroup, though it is unlikely to be able to serve as a single biomarker, given that heightened levels are seen in various conditions.

The data on OPN levels in various GNs are conflicting. In children, higher OPN levels were found in patients with IgAN and focal segmental glomerulosclerosis than in those with IgAN and minimal change disease [35]. In adults, OPN levels in those with MN and minimal change disease were normal or even reduced in those with IgAN [36]. Gang et al. [36] attributed their finding to the presence of thrombin-cleaved OPN fragments undetectable in their measurements. Building on that hypothesis, Kitagori et al. compared full and cleaved OPN (N fragments) in patients with LN, diabetic nephropathy, IgAN, and minimal change disease, finding no difference in full uOPN levels between the groups and increased levels of N fragments in patients with LN and diabetic nephropathy [37].

Our study focused on full-length uOPN but included samples from adult patients taken at two different time points. During their long follow-up, most patient received treatment with angiotensin-converting enzyme inhibitors and/or immunosuppressants, which might have influenced the results. Remission of disease, progressive fibrosis, or variance in the site of damage (glomerular vs. interstitial) could be factors responsible for discrepancies in OPN levels at baseline and follow-up [38]. However, given that control (protocol) biopsies were not performed, that hypothesis cannot be proved. The other explanation of this observation can be that uOPN is elevated only in the active form of IgAN and normalized to the same level as in other GN such as MN or LN, after the treatment. If it is true, OPN could become a very simple biomarker to be used, e.g., in an outpatient clinic, confirming active IgAN to be treated. This finding of our study must be validated.

**Figure 6:**
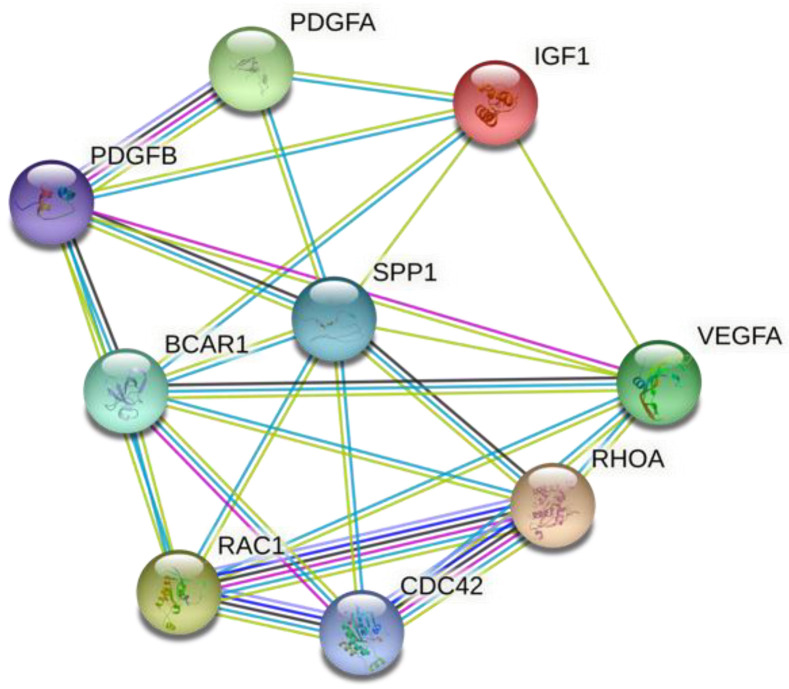
Proteins interacting with both osteopontin and platelets: BCAR1 = breast cancer anti-estrogen resistance protein 1; CDC42 = cell division control protein 42 homolog; IGF1 = insulin-like growth factor 1; PDGF = platelet-derived growth factor; RHOA = Ras’s homolog family member A; RAC1 = Ras-related C3 botulinum toxin substrate 1; SPP1 = signal peptide peptidase–osteopontin; VEGFA = vascular endothelial growth factor A. Assessed from the STRING-db database: https://string-db.org/ (accessed on 31 January 2022).

OPN is involved in many metabolic pathways, as shown in Figure 6 and Table 10. Because of the correlation with platelets, we have narrowed our variables to those two, and we have selected several proteins that might be goals for further investigation. Some of the functional pathways—for example, those connected to infectious diseases, particularly leishmaniosis—were also noted in another study concerning risk loci for IgAN [39]. So far, no single biomarker is likely to be diagnostic of a single GN, but in our opinion, a panel might bring the sensitivity and specificity that have long been sought.

Our study has several limitations. First, each GN was represented by a small sample. As our patient database evolves, those numbers will increase. For now, this research can be considered a preliminary study aimed at creating more interest in the topic. Second, further studies should include more proteins/markers or even the whole serum/urinary proteome. Third, OPN levels are likely to be linked to a histologic process and not to a specific GN. Correlation between OPN and a proteomic tissue analysis would be a valuable contribution and should be included in prospective studies. However, the need for the protocol to obtain control biopsies in patients during remission might be problematic. We did not correlate the OPN levels with kidney biopsy results for a few reasons: (1) each of studied glomerulonephritis has their own and completely different classification; (2) so, from this point of view the group was highly heterogenic, and (3) some biopsies were performed and evaluated in other centers by other pathologists that could influence the results. We summarized the biopsies result in Supplement, Tables S2–S4. We have not correlated uOPN levels with serum OPN levels or other biochemical results because the significance of OPN in other diseases is yet to be determined, and such a correlation effort would have unnecessarily complicated the analysis. In many patients, we also did not have access to certain data concerning previous history such as medications taken before study inclusion. Therefore, we decided against including these data in the study.

## 5. Conclusions

Given a growing burden of CKD, biomarker identification and validation have become an emerging issue. In our opinion, OPN should be included in further studies as a potential biomarker in nephrology. Based on our results, we are sure that ML should become a standard in biomarker research. As a supplement to ML, proteome databases can help place results in the context of numerous biologic pathways, pointing toward proteins that could be the next step in nephrology biomarker research.

## Figures and Tables

**Table 1:** Characteristics of the study participants at baseline (first sampling) and follow-up (second sampling).

Variable	Sampling	IgAN (*n =* 29)	LN (*n =* 18)	MN (*n =* 20)	Control (*n =* 13)	*p* Value
*Demographics* ^a,b^						
Age, years (avg ± SD)	1st	44 ± 12	43.74 ± 11.85	50.1 ± 14.09	44.38 ± 12.62	0.477
	2nd	48 ± 12	47.55 ± 12.26	51.86 ± 13.33	44.8 ± 14.81	0.799
Male (%)	1st	48	21	60	54	0.109
	2nd	50	0	50	60	0.023
BMI, kg/m^2^ (avg ± SD)	1st	26.3 ± 5.3	24.4 ± 4.6	26.1 ± 4.2	24.7 ± 2.0	0.541
	2nd	26.0 ± 4.9	24.0 ± 4.3	26.7 ± 4.1	24.8 ± 21.3	0.469
*Laboratory data (avg ± SD)* ^b^						
White blood cells (g/L)	1st	7.6 ± 2.3	6.4 ± 2.4	13.8 ± 8.2	5.7 ± 1.4	0.016
	2nd	7.9 ± 2.7	6.1 ± 1.9	7.1 ± 2.3	5.8 ± 2.1	0.114
Hemoglobin (g/dL)	1st	14.2 ± 1.5	12.7 ± 1.5	13.4 ± 1.9	14.2 ± 1.4	0.019
	2nd	13.7 ± 1.0	12.6 ± 1.2	13.1 ± 1.7	13.8 ± 1.0	0.095
Platelets (g/L)	1st	252.5 ± 59.6	255.1 ± 75.7	257.7 ± 62.4	232.1 ± 51.5	0.636
	2nd	244.2 ± 64.1	228.5 ± 94.2	237.4 ± 75.5	213 ± 46.3	0.824
Serum creatinine (mg/dL)	1st	1.3 ± 0.6	1.0 ± 0.3	1.2 ± 0.6	0.9 ± 0.1	0.155
	2nd	1.6 ± 0.9	1.0 ± 0.4	1.1 ± 0.5	0.9 ± 0.1	0.150
eGFR (mL/min × 1.73 m^2^)	1st	73.8 ± 31.3	80.3 ± 26.2	74.9 ± 29.0	94.4 ± 10.9	0.202
	2nd	61.0 ± 33.8	78.7 ± 31.6	74.3 ± 28.3	97.0 ± 14.1	0.111
Proteinuria (g/24 h)	1st	0.6 ± 0.6	0.8 ± 1.9	1.2 ± 1.4	n.a.	0.242
	2nd	0.9 ± 0.9	0.2 ± 0.1	0.6 ± 0.8	n.a.	0.052
*Comorbidities (n/n)*^a,^*						
Hypertension	1st	24/29	9/18	20/20	n.a.	<0.001
	2nd	15/18	5/11	20/14	n.a.	0.003
Coronary artery disease	1st	n.a.	1/18	5/20	n.a.	0.009
	2nd	n.a	1/11	2/14	n.a.	0.276
Atherosclerosis	1st	1/29	2/18	6/20	n.a.	0.026
	2nd	n.a.	n.a.	3/14	n.a.	n.a.
Anemia	1st	2/29	6/18	1/20	n.a.	0.015
	2nd	1/18	2/11	1/14	n.a.	0.495
Diabetes mellitus	1st	1/29	1/18	2/20	n.a.	0.634
	2nd	2/18	1/11	2/14	n.a.	0.919
Atrial fibrillation	1st	n.a.	n.a.	1/20	n.a.	n.a.
	2nd	n.a.	n.a.	1/14	n.a.	n.a.
Cancer	1st	1/29	1/18	2/20	n.a.	0.634
	2nd	1/18	1/11	2/14	n.a.	0.700
Autoimmune diseases (other)	1st	1/29	2/18	2/20	n.a.	0.546
	2nd	1/18	n.a.	2/14	n.a.	0.114
Infections	1st	2/29	6/18	3/20	n.a.	0.058
	2nd	1/18	1/11	1/14	n.a.	0.936
Tuberculosis	1st	1/29	1/18	1/20	n.a.	0.935
	2nd	n.a.	n.a.	1/14	n.a.	n.a.
Colon polyposis	1st	n.a.	1/18	1/20	n.a.	0.251
	2nd	n.a.	n.a.	n.a.	n.a.	n.a.
Dyslipidemia	1st	16/29	7/18	20/20	n.a.	<0.001
	2nd	12/18	6/11	14/14	n.a.	0.022
VTE disease	1st	1/18	2/18	5/20	n.a.	0.073
	2nd	n.a.	n.a.	3/14	n.a.	n.a.
Stroke/TIA	1st	1/29	1/18	1/20	n.a.	0.592
	2nd	n.a.	n.a.	n.a.	n.a.	n.a.
Thyroid diseases	1st	1/29	4/18	2/20	n.a.	0.123
	2nd	1/18	2/11	2/14	n.a.	0.548
*Medications (%)*^a,^*						
Immunosuppression	1st	10/29	17/18	15/20	n.a.	<0.001
	2nd	5/18	5/11	10/14	n.a.	0.002
Angiotensin-converting enzyme inhibitors	1st	24/29	14/18	14/20	n.a.	0.574
	2nd	14/18	8/11	9/14	n.a.	0.699
Angiotensin II receptor antagonists	1st	2/29	n.a.	9/20	n.a.	<0.001
	2nd	2/18	1/11	7/14	n.a.	0.015
Steroids	1st	10/29	14/18	14/20	n.a.	0.005
	2nd	5/18	9/11	11/14	n.a.	0.003

The level of significance was calculated using: ^a^—Chi-Squared test or ^b^—nonparametric Kruskal–Wallis test; * only within the glomerulopathies group, IgAN = immunoglobulin A nephropathy; LN = lupus nephritis; MN = membranous nephropathy; avg ± SD = average plus or minus the standard deviation; BMI = body mass index; eGFR = estimated glomerular filtration rate; n.a. = not available; VTE = venous thromboembolism; TIA = transient ischemic attack.

**Table 2:** Characteristics of the study participants during long-term clinical follow-up.

Variable	Clinical Laboratory Value (Mean ± SD)				
	IgAN (*n =* 29)	LN (*n* = 18)	MN (*n* = 20)	Control (*n* = 13)	*p* Value
BMI (kg/m^2^)	26.7 ± 5.3	24.8 ± 4.9	26.8 ± 4.4	24.7 ± 2.0	0.324
Serum creatinine (mg/dL)	1.3 ± 0.7	1.0 ± 0.3	1.2 ± 0.4	0.9 ± 0.13	0.029
eGFR (mL/min × 1.73 m^2^)	70.5 ± 31.0	83.6 ± 26.2	70.0 ± 21.9	97.1 ± 12.0	0.014
Hemoglobin (g/dL)	14.0 ± 1.22	12.6 ± 0.9	13.2 ± 1.6	14.0 ± 1.3	0.002
Platelets (g/L)	250.4 ± 56.5	246.3 ± 60.8	255.2 ± 61.2	237.7 ± 42.5	0.883
White blood cells (g/L)	8.0 ± 1.9	6.5 ± 2.3	8.7 ± 2.8	5.9 ± 1.4	<0.001
Proteinuria (g/24 h)	0.7 ± 0.6	0.4 ± 0.7	1.1 ± 1.4	n.a.	0.004
ΔeGFR (mL/min × 1.73 m^2^)	51.2 ± 31.9	42.7 ± 31.1	15.6 ± 21.2	−0.05 ± 5.6	<0.001
Months of total clinical follow-up	55.21 ± 14.97	46.22 ± 19.95	43.05 ± 12.09	23.64 ± 15.03	<0.001
Months of follow-up for OPN	33.72 ± 1.56	33.91 ± 1.7	19.36 ± 3.29	16.6 ± 2.07	<0.001

SD = standard deviation; IgAN = immunoglobulin A nephropathy; LN = lupus nephritis; MN = membranous nephropathy; BMI = body mass index; eGFR = estimated glomerular filtration rate; n.a. = not available. The level of significance was calculated using nonparametric Kruskal–Wallis test, *p* value was set as <0.05.

**Table 3:** Importance of variables that were not rejected by the Boruta algorithm for a Random Forest classifier that predicts the class of the patient.

Variable	Class				Mean
	Control	IgAN	LN	MN	
OPN	0.036	0.102	0.016	−0.013	0.042
WBC	0.067	0.028	0.051	0.010	0.033
eGFR	0.125	0.024	−0.009	−0.001	0.025
Hb	0.030	0.035	0.048	−0.031	0.019
CR	0.019	0.022	0.026	−0.017	0.012

IgAN = immunoglobulin A nephropathy; LN = lupus nephritis; MN = membranous nephropathy; OPN = osteopontin; WBC = white blood cells; eGFR = estimated glomerular filtration rate; Hb = hemoglobin; CR = creatinine.

**Table 4:** Average confusion matrix from 10 runs of a Random Forest classifier that predicts a patient’s glomerulopathy class using variables identified as relevant by Boruta.

		Control	IgAN	LN	MN	Class Error
**T**	Control	4.3	1.8	5.7	1.2	0.67
**R**	IgAN	2.1	19.9	2.6	4.4	0.31
**U**	LN	1.9	3.0	8.2	4.9	0.54
**E**	MN	2.8	6.3	6.6	4.3	0.78

IgAN = immunoglobulin A nephropathy; LN = lupus nephritis; MN = membranous nephropathy.

**Table 5:** Importance of variables that were not rejected by the Boruta algorithm for a Random Forest classifier that discerns IgAN from all other classes.

Variable	Non-IgAN	IgAN	Mean
OPN	0.065	0.130	0.087
WBC	0.029	0.019	0.025
eGFR	0.015	0.023	0.018
CR	0.016	0.022	0.017
Hb	0.005	0.038	0.017

IgAN = immunoglobulin A nephropathy; OPN = osteopontin; WBC = white blood cells; eGFR = estimated glomerular filtration rate; Hb = hemoglobin; CR = creatinine.

**Table 6:** Predicted average confusion matrix from 10 runs of a Random Forest classifier that discerns IgAN from other glomerulopathy classes using variables identified as relevant by Boruta.

	Non-IgAN	IgAN	Class Error
**Non-IgAN**	44.2	6.8	0.13
**IgAN**	12.5	16.5	0.43

IgAN = immunoglobulin A nephropathy.

**Table 7:** Importance of variables that were not rejected by the Boruta algorithm for a Random Forest classifier that predicts the glomerulopathy class of the patient.

		Control	IgAN	LN	MN	Mean
	OPN	−0.006	**0.132**	−0.001	−0.008	0.028
T	WBC	0.003	0.039	0.051	−0.014	0.016
R	Px3	−0.018	9.1 × 10^−5^	0.056	0.020	0.016
U	Hb	−0.010	0.008	0.037	−0.037	0.015
E	eGFR	0.068	0.018	−0.008	0.010	0.015
	Px1	0.007	0.018	−0.004	−0.002	0.005
	Px4	−0.049	4.8 × 10^−4^	0.054	−0.005	0.004

IgAN = immunoglobulin A nephropathy; LN = lupus nephritis; MN = membranous nephropathy; OPN = osteopontin; eGFR = estimated glomerular filtration rate; WBC = white blood cells; Hb = hemoglobin; Px = peroxiredoxin.

**Table 8:** Average confusion matrix from 10 runs of a Random Forest classifier built on the reduced data set consisting of 53 patients.

		Control	IgAN	LN	MN	Class Error
T	Ctrl	0.0	0.0	3.0	4.0	1.0
R	IgAN	1.0	9.8	1.0	4.2	0.38
U	LN	0.1	1.0	6.9	4.0	0.42
E	MN	2.8	3.9	4.3	7.0	0.61

IgAN = immunoglobulin A nephropathy; LN = lupus nephritis; MN = membranous nephropathy.

**Table 9:** Spearman correlation analysis of clinical parameters and osteopontin levels at follow-up.

Parameter	IgAN			LN			MN			Control		
	*R*	*R* ^2^	*p* Value	*R*	*R* ^2^	*p* Value	*R*	*R* ^2^	*p* Value	*R*	*R* ^2^	*p* Value
Age (years)	−0.096	0.009	0.705	0.346	0.120	0.297	−0.236	0.056	0.416	0.300	0.090	0.624
BMI (kg/m^2^)	−0.282	0.079	0.257	0.582	0.339	0.060	−0.020	0.000	0.946	0.100	0.010	0.873
WBC (g/L)	0.007	1 × 10^−4^	0.977	−0.091	0.008	0.790	−0.051	0.003	0.864	0.300	0.090	0.624
Hb (g/dL)	−0.088	0.008	0.729	0.014	0.000	0.968	0.106	0.011	0.719	0.900	0.810	**0.037**
PLT (g/L)	0.483	0.234	**0.042**	0.055	0.003	0.873	0.305	0.093	0.288	0.200	0.040	0.747
Serum CR (mg/dL)	−0.358	0.128	0.145	−0.182	0.033	0.593	−0.248	0.062	0.392	1.000	1.000	n.a.
eGFR (mL/min × 1.73 m^2^)	0.377	0.142	0.123	0.000	0.000	1.000	0.385	0.148	0.175	−0.300	0.090	0.624
Proteinuria (g/24 h)	0.126	0.016	0.618	−0.355	0.126	0.284	−0.544	0.296	0.055	n.a.	n.a.	n.a.

IgAN = immunoglobulin A nephropathy; LN = lupus nephritis; MN = membranous nephropathy; BMI = body mass index; WBC = white blood cells; Hb = hemoglobin; PLT = platelets; CR = creatinine; eGFR = estimated glomerular filtration rate. n.a. = not available.

**Table 10:** Functional analysis of selected genes linked to *SPP1* (OPN) [24,25].

Category and Term ^1^	Gene Count		Strength	False Discovery Rate ^2^	Term Identifier
	Observed	Background			
*Diseases (gene associations)*					
Disease of cellular proliferation	25	1012	0.82	1.12 × 10^−10^	DOID:14566
Cancer	23	895	0.83	3.58 × 10^−10^	DOID:162
Ischemia	5	23	1.76	5.31 × 10^−5^	DOID:326
Vascular disease	9	223	1.03	1.6 × 10^−4^	DOID:178
*Gene ontology (molecular function)*					
Platelet-derived growth factoreceptor binding	7	15	2.09	6.86 × 10^−10^	GO:0005161
Phosphatidylinositol 3–kinase binding	6	30	1.72	1.08 × 10^−6^	GO:0043548
Growth factor activity	10	161	1.22	2.98 × 10^−7^	GO:0008083
Integrin binding	9	147	1.21	1.61 × 10^−6^	GO:0005178
Signaling receptor binding	44	1581	0.87	2.91 × 10^−25^	GO:0005102
Cell adhesion molecule binding	15	538	0.87	5.26 × 10^−7^	GO:0050839
Enzyme activator activity	12	520	0.79	8.58 × 10^−5^	GO:0008047
*Gene ontology (biologic process)*					
Signal transduction	52	2741	0.7	2.93 × 10^−24^	HSA-162582
Immune system	40	1956	0.73	1.23 × 10^−17^	HSA-168256
Signaling by VEGF	16	106	1.6	1.54 × 10^−17^	HSA-194138
VEGFA–VEGFR2 pathway	15	97	1.61	1.81 × 10^−16^	HSA-4420097
Signaling by interleukins	20	440	1.08	2.90 × 10^−13^	HSA-449147
Innate immune system	27	1025	0.84	3.29 × 10^−13^	HSA-168249
Cytokine signaling in immune system	22	681	0.93	6.08 × 10^−12^	HSA-1280215
Integrin signaling	8	27	1.89	3.46 × 10^−10^	HSA-354192
Platelet activation, signaling, and aggregation	35	260	1.55	1.58 × 10^−40^	HSA-76002
Platelet degranulation	17	127	1.55	7.74 × 10^−18^	HSA-114608
Platelet aggregation (plug formation)	9	39	1.79	8.88 × 10^−11^	HSA-76009
Signaling by PDGF	8	58	1.56	5.24 × 10^−8^	HSA-186797
Factors involved in megakaryocyte development and platelet production	10	154	1.23	1.97 × 10^−7^	HSA-983231
Infectious disease	25	826	0.9	2.80 × 10^−13^	HSA-5663205
Leishmania infection	14	249	1.17	5.10 × 10^−10^	HSA-9658195
Regulation of actin dynamics for phagocytic cup formation	9	62	1.58	2.76 × 10^−9^	HSA-2029482

DOID = disease ontology identifier; GO = genetic ontology; HAS = molecular pathway identifier (*Homo sapiens*). ^1^ For each category, selected terms are shown. ^2^ Values less than 0.0001 are considered statistically significant.

## Data Availability

Data are available on request.

## Supplementary Materials

The following supporting information can be downloaded at https://www.mdpi.com/article/10.3390/biomedicines10040734/s1, File S1: machine learning scripts applied in the R software; File S2: list of selected proteins from the STRING database; Table S1: correlations of OPN with clinical parameters and long-term clinical follow-up; Tables S2–S4: histopathological classification of study participants.

## References

[1] Centers for Disease Control and Prevention (2021). Chronic Kidney Disease in the United States, 2021.

[2] Pac M., Krata N., Moszczuk B., Wyczałkowska-Tomasik A., Kaleta B., Foroncewicz B., Rudnicki W., Pączek L., Mucha K. (2021). NR3C1 Glucocorticoid Receptor Gene Polymorphisms Are Associated with Membranous and IgA Nephropathies. Cells.

[3] Xie J., Liu L., Mladkova N., Li Y., Ren H., Wang W., Cui Z., Lin L., Hu X., Yu X. (2020). The genetic architecture of membranous nephropathy and its potential to improve non-invasive diagnosis. Nat. Commun..

[4] Kaimori J.Y., Takenaka M., Nagasawa Y., Nakajima H., Izumi M., Akagi Y., Imai E., Hori M. (2002). Quantitative analyses of osteopontin mRNA expression in human proximal tubules isolated from renal biopsy tissue sections of minimal change nephrotic syndrome and IgA glomerulonephropathy patients. Am. J. Kidney Dis..

[5] Krata N., Foroncewicz B., Zagożdżon R., Moszczuk B., Zielenkiewicz M., Pączek L., Mucha K. (2021). Peroxiredoxins as Markers of Oxidative Stress in IgA Nephropathy, Membranous Nephropathy and Lupus Nephritis. Arch. Immunol. Ther. Exp..

[6] Moszczuk B., Kiryluk K., Pączek L., Mucha K. (2021). Membranous Nephropathy: From Research Bench to Personalized Care. J. Clin. Med..

[7] Icer M.A., Gezmen-Karadag M. (2018). The multiple functions and mechanisms of osteopontin. Clin. Biochem..

[8] Cheema B.S., Iyengar S., Sharma R., Kohli H.S., Bhansali A., Khullar M. (2015). Association between Osteopontin Promoter Gene Polymorphisms and Haplotypes with Risk of Diabetic Nephropathy. J. Clin. Med..

[9] Kaleta B., Krata N., Zagożdżon R., Mucha K. (2019). Osteopontin Gene Polymorphism and Urinary OPN Excretion in Patients with Immunoglobulin A Nephropathy. Cells.

[10] Xu C.X., Zhang Y.L., Huang X.Y., Han F., Jin Z.K., Tian P.X., Dou M. (2021). Prediction of acute renal allograft rejection by combined HLA-G 14-bp insertion/deletion genotype analysis and detection of kidney injury molecule-1 and osteopontin in the peripheral blood. Transpl. Immunol..

[11] Kohl K., Herzog E., Dickneite G., Pestel S. (2020). Evaluation of urinary biomarkers for early detection of acute kidney injury in a rat nephropathy model. J. Pharmacol. Toxicol. Methods.

[12] Wu J., Zhao J., Zhao Z., Jin S., Yu Q. (2021). Significance of TRPV5 and OPN biomarker levels in clinical diagnosis of patients with early urinary calculi. Am. J. Transl. Res..

[13] Wirestam L., Enocsson H., Skogh T., Padyukov L., Jönsen A., Urowitz M.B., Gladman D.D., Romero-Diaz J., Bae S.C., Fortin P.R. (2019). Osteopontin and Disease Activity in Patients with Recent-onset Systemic Lupus Erythematosus: Results from the SLICC Inception Cohort. J. Rheumatol..

[14] Gordin D., Forsblom C., Panduru N.M., Thomas M.C., Bjerre M., Soro-Paavonen A., Tolonen N., Sandholm N., Flyvbjerg A., Harjutsalo V. (2014). Osteopontin is a strong predictor of incipient diabetic nephropathy, cardiovascular disease, and all-cause mortality in patients with type 1 diabetes. Diabetes Care.

[15] Spinelli F.R., Garufi C., Truglia S., Pacucci V.A., Morello F., Miranda F., Perricone C., Ceccarelli F., Valesini G., Conti F. (2019). The role of osteopontin as a candidate biomarker of renal involvement in systemic lupus erythematosus. Clin. Exp. Rheumatol..

[16] McGrogan A., Franssen C.F., de Vries C.S. (2011). The incidence of primary glomerulonephritis worldwide: A systematic review of the literature. Nephrol. Dial. Transplant..

[17] Breiman L. (2001). Random Forests. Mach. Learn..

[18] Kursa M.B., Jankowski A., Rudnicki W.R. (2010). Boruta—A System for Feature Selection. Fundam. Inform..

[19] Liaw A., Wiener M. (2001). Classification and Regression by RandomForest. Forest.

[20] Kursa M.B., Rudnicki W.R. (2010). Feature Selection with the Boruta Package. J. Stat. Softw..

[21] R Core Team (2018). R: A Language and Environment for Statistical Computing.

[22] Helluin O., Chan C., Vilaire G., Mousa S., DeGrado W.F., Bennett J.S. (2000). The activation state of alphavbeta 3 regulates platelet and lymphocyte adhesion to intact and thrombin-cleaved osteopontin. J. Biol. Chem..

[23] https://www.uniprot.org/uniprot/P10451.

[24] Szklarczyk D., Gable A.L., Nastou K.C., Lyon D., Kirsch R., Pyysalo S., Doncheva N.T., Legeay M., Fang T., Bork P. (2021). The STRING database in 2021: Customizable protein-protein networks, and functional characterization of user-uploaded gene/measurement sets. Nucleic Acids Res..

[25] Consortium T.U. (2020). UniProt: The universal protein knowledgebase in 2021. Nucleic Acids Res..

[26] Kanehisa M., Goto S. (2000). KEGG: Kyoto encyclopedia of genes and genomes. Nucleic Acids Res..

[27] Haynes W., Dubitzky W., Wolkenhauer O., Cho K.-H., Yokota H. (2013). Benjamini–Hochberg Method. Encyclopedia of Systems Biology.

[28] Boutet A., Madhavan R., Elias G.J.B., Joel S.E., Gramer R., Ranjan M., Paramanandam V., Xu D., Germann J., Loh A. (2021). Predicting optimal deep brain stimulation parameters for Parkinson’s disease using functional MRI and machine learning. Nat. Commun..

[29] Iwendi C., Bashir A.K., Peshkar A., Sujatha R., Chatterjee J.M., Pasupuleti S., Mishra R., Pillai S., Jo O. (2020). COVID-19 Patient Health Prediction Using Boosted Random Forest Algorithm. Front. Public Health.

[30] Jumper J., Evans R., Pritzel A., Green T., Figurnov M., Ronneberger O., Tunyasuvunakool K., Bates R., Žídek A., Potapenko A. (2021). Highly accurate protein structure prediction with AlphaFold. Nature.

[31] Vamathevan J., Clark D., Czodrowski P., Dunham I., Ferran E., Lee G., Li B., Madabhushi A., Shah P., Spitzer M. (2019). Applications of machine learning in drug discovery and development. Nat. Rev. Drug Discov..

[32] Fernández-Delgado M., Cernadas E., Barro S., Amorim D. (2014). Do we need hundreds of classifiers to solve real world classification problems?. J. Mach. Learn. Res..

[33] Rudnicki W., Wrzesień M., Paja W. (2015). All Relevant Feature Selection Methods and Applications. Stud. Comput. Intell..

[34] Li Q., Fan Q.-L., Han Q.-X., Geng W.-J., Zhao H.-H., Ding X.-N., Yan J.-Y., Zhu H.-Y. (2020). Machine learning in nephrology: Scratching the surface. Chin. Med. J..

[35] Wasilewska A., Taranta-Janusz K., Kuroczycka-Saniutycz E., Zoch-Zwierz W. (2011). Urinary OPN excretion in children with glomerular proteinuria. Adv. Med. Sci..

[36] Gang X., Ueki K., Kon S., Maeda M., Naruse T., Nojima Y. (2001). Reduced urinary excretion of intact osteopontin in patients with IgA nephropathy. Am. J. Kidney Dis..

[37] Kitagori K., Yoshifuji H., Oku T., Sasaki C., Miyata H., Mori K.P., Nakajima T., Ohmura K., Kawabata D., Yukawa N. (2016). Cleaved Form of Osteopontin in Urine as a Clinical Marker of Lupus Nephritis. PLoS ONE.

[38] Nagao T., Okura T., Irita J., Jotoku M., Enomoto D., Desilva V.R., Miyoshi K.-I., Kurata M., Matsui Y., Uede T. (2012). Osteopontin plays a critical role in interstitial fibrosis but not glomerular sclerosis in diabetic nephropathy. Nephron Extra.

[39] Kiryluk K., Li Y., Scolari F., Sanna-Cherchi S., Choi M., Verbitsky M., Fasel D., Lata S., Prakash S., Shapiro S. (2014). Discovery of new risk loci for IgA nephropathy implicates genes involved in immunity against intestinal pathogens. Nat. Genet..

