# Supplementary material for: Osteopontin—A Potential Biomarker for IgA Nephropathy: Machine Learning Application
Source: Biomedicines. 2022 Mar 22;10(4):734. doi: 10.3390/biomedicines10040734 (PMC9025015; doi:10.3390/biomedicines10040734)
Supplement: Supplementary file 1 [file biomedicines-10-00734-s001.zip › File S1]

R Notebook


Code 

- Show All Code
- Hide All Code
- Download Rmd

# R Notebook


```
library("openxlsx")
read.xlsx("Nowy_all.xlsx",colNames = T) -> X
library(randomForest)
library(Boruta)
X$Group <- as.factor(X$Group)
X$Gender <- as.factor(X$Gender)
X$eGFRgroup <- as.factor(X$eGFRgroup)
```


Type of condition as a decision variable First part - we take only these variables with complete coverage of all patients Then we remove patient ID and ilness type from descriptors. Finally, we take onto account only the averages of measurements for all variables, and rename variables.


```
Mask.na <- vector("logical",dim(X)[2])
for (i in 1:dim(X)[2]) Mask.na[i] <- sum(is.na(X[,i]))>0
X.new <- X[,!Mask.na]
X.new <- X.new[,-1]
X.new.x <- X.new[,-1]
X.new.x <- X.new.x[,-2]
print(dim(X.new.x))
```


```
[1] 80  8
```


```
print(names(X.new.x))
```


```
[1] "Gender"  "BMI_m"   "creat_m" "eGFR_m"  "HB_m"    "PLT_m"   "WBC_m"   "OPN1"
```


```
names(X.new.x) <- c("Gndr","BMI","CR","eGFR","Hb","PLT","WBC","OPN")
print(names(X.new.x))
```


```
[1] "Gndr" "BMI"  "CR"   "eGFR" "Hb"   "PLT"  "WBC"  "OPN"
```


Now we perform tests First we simply test which variables contribute for prediction of ilness type. We set a seed to a constant value to get results that are exaclty reproducible.


```
Y.new.4c <- X.new[,1]
randomForest(x=X.new.x,y=as.factor(as.character(Y.new.4c)),importance = T) -> rf.4c 
print(rf.4c)
```


```
Call:
 randomForest(x = X.new.x, y = as.factor(as.character(Y.new.4c)),      importance = T) 
               Type of random forest: classification
                     Number of trees: 500
No. of variables tried at each split: 2

        OOB estimate of  error rate: 55%
Confusion matrix:
     Ctrl IgAN LN MN class.error
Ctrl    6    1  6  0   0.5384615
IgAN    3   18  4  4   0.3793103
LN      3    2  8  5   0.5555556
MN      1   11  4  4   0.8000000
```


```
print(rf.4c)
```


```
Call:
 randomForest(x = X.new.x, y = as.factor(as.character(Y.new.4c)),      importance = T) 
               Type of random forest: classification
                     Number of trees: 500
No. of variables tried at each split: 2

        OOB estimate of  error rate: 55%
Confusion matrix:
     Ctrl IgAN LN MN class.error
Ctrl    6    1  6  0   0.5384615
IgAN    3   18  4  4   0.3793103
LN      3    2  8  5   0.5555556
MN      1   11  4  4   0.8000000
```


```
set.seed(2022)
Boruta(x=X.new.x,y=as.factor(as.character(Y.new.4c)),doTrace = 1) -> Bor.4c
```


```
After 10 iterations, +0.33 secs: 
 confirmed 1 attribute: OPN;
 rejected 2 attributes: BMI, PLT;
 still have 5 attributes left.

After 14 iterations, +0.48 secs: 
 confirmed 2 attributes: Hb, WBC;
 rejected 1 attribute: Gndr;
 still have 2 attributes left.

After 17 iterations, +0.56 secs: 
 confirmed 1 attribute: eGFR;
 still have 1 attribute left.
```


```
plot(Bor.4c)
```


```
Bor.4c$finalDecision != "Rejected" -> Bor.4c.selected
print(Bor.4c$finalDecision)
```


```
     Gndr       BMI        CR      eGFR        Hb       PLT       WBC       OPN 
 Rejected  Rejected Tentative Confirmed Confirmed  Rejected Confirmed Confirmed 
Levels: Tentative Confirmed Rejected
```


```
print(Bor.4c.selected)
```


```
[1] FALSE FALSE  TRUE  TRUE  TRUE FALSE  TRUE  TRUE
```


```
rf.4c.list <- list()
for (i in 1:10) {
  randomForest(x=X.new.x[,Bor.4c.selected],y=as.factor(as.character(Y.new.4c)),importance = T) -> rf.4c.list[[i]]
  #print(rf.4c.list[[i]])
}
rf.4c.avg.conf  <- rf.4c.list[[1]]$confusion
for (i in 2:10) rf.4c.avg.conf <- rf.4c.avg.conf + rf.4c.list[[i]]$confusion
rf.4c.avg.conf <- rf.4c.avg.conf/10
print(rf.4c.avg.conf)
```


```
     Ctrl IgAN  LN  MN class.error
Ctrl  4.3  1.8 5.7 1.2   0.6692308
IgAN  2.1 19.9 2.6 4.4   0.3137931
LN    1.9  3.0 8.2 4.9   0.5444444
MN    2.8  6.3 6.6 4.3   0.7850000
```


```
rf.4c.avg.oob  <- rf.4c.list[[1]]$err.rate[500,1]
for (i in 2:10) rf.4c.avg.oob <- rf.4c.avg.oob + rf.4c.list[[i]]$err.rate[500,1]
rf.4c.avg.oob <- rf.4c.avg.oob/10
print(rf.4c.avg.oob)
```


```
    OOB 
0.54125
```


```
rf.4c.avg.imp  <- rf.4c.list[[1]]$importance
for (i in 2:10) rf.4c.avg.imp <- rf.4c.avg.imp + rf.4c.list[[i]]$importance
rf.4c.avg.imp <- rf.4c.avg.imp/10
print(rf.4c.avg.imp[order(rf.4c.avg.imp[,5],decreasing = T),1:5])
```


```
           Ctrl       IgAN          LN            MN MeanDecreaseAccuracy
OPN  0.03577032 0.10186074  0.01571603 -0.0125591159           0.04172560
WBC  0.06659587 0.02820421  0.05158426  0.0104323027           0.03290171
eGFR 0.12529627 0.02482051 -0.00892767 -0.0007088251           0.02512170
Hb   0.03029579 0.03510571  0.04807556 -0.0308547131           0.01978039
CR   0.01903197 0.02184379  0.02606784 -0.0172951743           0.01231813
```


Randomisation experiment


```
rand.rf <- list(NULL)
 
for (i in 1:10) {
  Y.rand <- sample(Y.new.4c,80,replace = F)
  randomForest(x=X.new.x[,Bor.4c.selected],y=as.factor(as.character(Y.rand)),importance = T) -> rand.rf[[i]]
}
rf.rand.conf  <- rand.rf[[1]]$confusion
for (i in 2:10) rf.rand.conf <- rf.rand.conf + rand.rf[[i]]$confusion
rf.rand.conf <- rf.rand.conf/10
print(rf.rand.conf)
```


```
     Ctrl IgAN  LN  MN class.error
Ctrl  0.6  7.6 2.2 2.6   0.9538462
IgAN  3.3 14.7 5.9 5.1   0.4931034
LN    1.6  8.8 2.7 4.9   0.8500000
MN    2.1  9.2 5.3 3.4   0.8300000
```


```
rf.rand.oob  <- rand.rf[[1]]$err.rate[500,1]
for (i in 2:10) rf.rand.oob <- rf.rand.oob + rand.rf[[i]]$err.rate[500,1]
rf.rand.oob <- rf.rand.oob/10
print(rf.rand.oob)
```


```
   OOB 
0.7325
```


```
rf.rand.imp  <- rand.rf[[1]]$importance
for (i in 2:10) rf.rand.imp <- rf.rand.imp + rand.rf[[i]]$importance
rf.rand.imp <- rf.rand.imp/10
print(rf.rand.imp[order(rf.rand.imp[,5],decreasing = T),1:5])
```


```
              Ctrl          IgAN            LN           MN MeanDecreaseAccuracy
CR   -0.0043661111  0.0144485865  0.0009366556 -0.012124182         0.0014010649
OPN  -0.0020534127  0.0102581280 -0.0044699739 -0.003943211         0.0009870387
Hb   -0.0062820635  0.0105248666 -0.0040405744 -0.013044936        -0.0011485378
eGFR -0.0051550000  0.0050495477 -0.0047332989 -0.005764466        -0.0016766310
WBC  -0.0007276984 -0.0004253573 -0.0041263825 -0.001363548        -0.0017083690
```


We can see, that with randomised version of variables we get random classifier with no relevant variables.

Now we check whether we can discern IgAN from other classes


```
Y.new.igan <- X.new[,1] == "IgAN"
set.seed(2022)
randomForest(x=X.new.x,y=as.factor(Y.new.igan),importance = T) -> rf.igan 
print(rf.igan)
```


```
Call:
 randomForest(x = X.new.x, y = as.factor(Y.new.igan), importance = T) 
               Type of random forest: classification
                     Number of trees: 500
No. of variables tried at each split: 2

        OOB estimate of  error rate: 23.75%
Confusion matrix:
      FALSE TRUE class.error
FALSE    44    7   0.1372549
TRUE     12   17   0.4137931
```


```
print(rf.igan)
```


```
Call:
 randomForest(x = X.new.x, y = as.factor(Y.new.igan), importance = T) 
               Type of random forest: classification
                     Number of trees: 500
No. of variables tried at each split: 2

        OOB estimate of  error rate: 23.75%
Confusion matrix:
      FALSE TRUE class.error
FALSE    44    7   0.1372549
TRUE     12   17   0.4137931
```


```
Boruta(x=X.new.x,y=as.factor(as.character(Y.new.igan)),doTrace = 1) -> Bor.igan
```


```
After 10 iterations, +0.26 secs: 
 confirmed 1 attribute: OPN;
 rejected 3 attributes: BMI, Gndr, PLT;
 still have 4 attributes left.

After 14 iterations, +0.36 secs: 
 confirmed 2 attributes: Hb, WBC;
 still have 2 attributes left.

After 21 iterations, +0.52 secs: 
 confirmed 2 attributes: CR, eGFR;
 no more attributes left.
```


```
plot(Bor.igan)
```


```
Bor.igan$finalDecision != "Rejected" -> Bor.igan.selected
print(Bor.igan$finalDecision)
```


```
     Gndr       BMI        CR      eGFR        Hb       PLT       WBC       OPN 
 Rejected  Rejected Confirmed Confirmed Confirmed  Rejected Confirmed Confirmed 
Levels: Tentative Confirmed Rejected
```


```
print(Bor.igan.selected)
```


```
[1] FALSE FALSE  TRUE  TRUE  TRUE FALSE  TRUE  TRUE
```


```
rf.igan.list <- list()
for (i in 1:10) {
  randomForest(x=X.new.x[,Bor.igan.selected],y=as.factor(as.character(Y.new.igan)),importance = T) -> rf.igan.list[[i]]
  #print(rf.igan.list[[i]])
}
rf.igan.avg.conf  <- rf.igan.list[[1]]$confusion
for (i in 2:10) rf.igan.avg.conf <- rf.igan.avg.conf + rf.igan.list[[i]]$confusion
rf.igan.avg.conf <- rf.igan.avg.conf/10
print(rf.igan.avg.conf)
```


```
      FALSE TRUE class.error
FALSE  44.2  6.8   0.1333333
TRUE   12.5 16.5   0.4310345
```


```
rf.igan.avg.oob  <- rf.igan.list[[1]]$err.rate[500,1]
for (i in 2:10) rf.igan.avg.oob <- rf.igan.avg.oob + rf.igan.list[[i]]$err.rate[500,1]
rf.igan.avg.oob <- rf.igan.avg.oob/10
print(rf.igan.avg.oob)
```


```
    OOB 
0.24125
```


```
rf.igan.avg.imp  <- rf.igan.list[[1]]$importance
for (i in 2:10) rf.igan.avg.imp <- rf.igan.avg.imp + rf.igan.list[[i]]$importance
rf.igan.avg.imp <- rf.igan.avg.imp/10
print(rf.igan.avg.imp[order(rf.igan.avg.imp[,3],decreasing = T),1:3])
```


```
           FALSE       TRUE MeanDecreaseAccuracy
OPN  0.065197620 0.13039281           0.08756617
WBC  0.029636558 0.01889549           0.02545203
eGFR 0.015549772 0.02310244           0.01788524
CR   0.014638087 0.02166257           0.01685484
Hb   0.005095269 0.03833387           0.01660957
```


Yes, we can. Five variables contribute. By far most relevant variable is OPN.

Now we will repeat the analyses with data set limited to patients who have PRDXx measurements, and all variables that were recorded for these patients will be included.


```
Mask.prdx <- !is.na(X$PRDX1)
X.prdx <- X[Mask.prdx,]
set.seed(2022)
Mask.na <- vector("logical",dim(X.prdx)[2])
for (i in 1:dim(X)[2]) Mask.na[i] <- sum(is.na(X.prdx[,i]))>0
X.prdx <- X.prdx[,!Mask.na]
X.prdx.x <- X.prdx[,c(-1,-2,-4,-5,-8,-9,-17)]
# we remove all non mean measurements 
X.prdx.x.m <- X.prdx.x[,c(-2,-3)]
names(X.prdx.x.m)  <- c("Gndr","BMI","CR","eGFR","Hb","PLT","WBC","OPN","Px1","Px2","Px3","Px4","Px5")
Y.prdx <- X.prdx$Group
randomForest(x=X.prdx.x,y=as.factor(Y.prdx),importance = T) -> rf.prdx 
print(rf.prdx)
```


```
Call:
 randomForest(x = X.prdx.x, y = as.factor(Y.prdx), importance = T) 
               Type of random forest: classification
                     Number of trees: 500
No. of variables tried at each split: 3

        OOB estimate of  error rate: 52.83%
Confusion matrix:
     Ctrl IgAN LN MN class.error
Ctrl    1    0  3  3   0.8571429
IgAN    1    9  1  5   0.4375000
LN      0    1  5  6   0.5833333
MN      2    3  3 10   0.4444444
```


```
randomForest(x=X.prdx.x.m,y=as.factor(Y.prdx),importance = T) -> rf.prdx.m
print(rf.prdx.m)
```


```
Call:
 randomForest(x = X.prdx.x.m, y = as.factor(Y.prdx), importance = T) 
               Type of random forest: classification
                     Number of trees: 500
No. of variables tried at each split: 3

        OOB estimate of  error rate: 56.6%
Confusion matrix:
     Ctrl IgAN LN MN class.error
Ctrl    0    0  4  3   1.0000000
IgAN    1   10  1  4   0.3750000
LN      2    1  5  4   0.5833333
MN      3    4  3  8   0.5555556
```


```
Boruta(x=X.prdx.x,y=as.factor(as.character(Y.prdx)),doTrace = 1) -> Bor.prdx
```


```
After 11 iterations, +0.29 secs: 
 rejected 5 attributes: BMI_m, eGFR1, Gender, PLT_m, PRDX2;
 still have 10 attributes left.

After 15 iterations, +0.38 secs: 
 confirmed 1 attribute: OPN1;
 rejected 1 attribute: PRDX5;
 still have 8 attributes left.

After 22 iterations, +0.54 secs: 
 confirmed 1 attribute: HB_m;
 still have 7 attributes left.

After 31 iterations, +0.72 secs: 
 rejected 1 attribute: PRDX1;
 still have 6 attributes left.

After 36 iterations, +0.83 secs: 
 rejected 1 attribute: creat_m;
 still have 5 attributes left.

After 52 iterations, +1.2 secs: 
 confirmed 1 attribute: PRDX3;
 still have 4 attributes left.

After 87 iterations, +1.9 secs: 
 confirmed 1 attribute: WBC_m;
 still have 3 attributes left.
```


```
plot(Bor.prdx)
```


```
Bor.prdx$finalDecision != "Rejected" -> Bor.prdx.selected
print(Bor.prdx$finalDecision)
```


```
   Gender     eGFR1     eGFR3     BMI_m   creat_m    eGFR_m 
 Rejected  Rejected Tentative  Rejected  Rejected Tentative 
     HB_m     PLT_m     WBC_m      OPN1     PRDX1     PRDX2 
Confirmed  Rejected Confirmed Confirmed  Rejected  Rejected 
    PRDX3     PRDX4     PRDX5 
Confirmed Tentative  Rejected 
Levels: Tentative Confirmed Rejected
```


```
print(Bor.prdx.selected)
```


```
 [1] FALSE FALSE  TRUE FALSE FALSE  TRUE  TRUE FALSE  TRUE
[10]  TRUE FALSE FALSE  TRUE  TRUE FALSE
```


```
#
# Smaller data set
#
Boruta(x=X.prdx.x.m,y=as.factor(as.character(Y.prdx)),doTrace = 1) -> Bor.prdx.m
```


```
After 11 iterations, +0.28 secs: 
 rejected 2 attributes: Gndr, PLT;
 still have 11 attributes left.

After 15 iterations, +0.37 secs: 
 confirmed 1 attribute: OPN;
 rejected 2 attributes: BMI, Px2;
 still have 8 attributes left.

After 21 iterations, +0.49 secs: 
 confirmed 1 attribute: Hb;
 still have 7 attributes left.

After 25 iterations, +0.6 secs: 
 rejected 1 attribute: Px5;
 still have 6 attributes left.

After 36 iterations, +0.83 secs: 
 confirmed 2 attributes: eGFR, Px3;
 still have 4 attributes left.

After 54 iterations, +1.2 secs: 
 rejected 1 attribute: CR;
 still have 3 attributes left.

After 65 iterations, +1.5 secs: 
 confirmed 1 attribute: WBC;
 still have 2 attributes left.
```


```
plot(Bor.prdx.m)
```


```
Bor.prdx.m$finalDecision != "Rejected" -> Bor.prdx.m.selected
print(Bor.prdx.m$finalDecision)
```


```
     Gndr       BMI        CR      eGFR        Hb       PLT 
 Rejected  Rejected  Rejected Confirmed Confirmed  Rejected 
      WBC       OPN       Px1       Px2       Px3       Px4 
Confirmed Confirmed Tentative  Rejected Confirmed Tentative 
      Px5 
 Rejected 
Levels: Tentative Confirmed Rejected
```


```
print(Bor.prdx.m.selected)
```


```
 [1] FALSE FALSE FALSE  TRUE  TRUE FALSE  TRUE  TRUE  TRUE
[10] FALSE  TRUE  TRUE FALSE
```


```
X.prdx.x.m.imp <- X.prdx.x.m[,Bor.prdx.m.selected]
Boruta(x=X.prdx.x.m.imp,y=as.factor(as.character(Y.prdx)),doTrace = 1) -> Bor.prdx.m.imp
```


```
After 10 iterations, +0.22 secs: 
 confirmed 2 attributes: Hb, OPN;
 still have 5 attributes left.

After 17 iterations, +0.37 secs: 
 confirmed 1 attribute: Px3;
 still have 4 attributes left.

After 26 iterations, +0.56 secs: 
 confirmed 1 attribute: WBC;
 still have 3 attributes left.

After 29 iterations, +0.62 secs: 
 confirmed 1 attribute: eGFR;
 still have 2 attributes left.
```


```
plot(Bor.prdx.m.imp)
```


```
Bor.prdx.m.imp$finalDecision != "Rejected" -> Bor.prdx.m.imp.selected
print(Bor.prdx.m.imp$finalDecision)
```


```
     eGFR        Hb       WBC       OPN       Px1       Px3 
Confirmed Confirmed Confirmed Confirmed Tentative Confirmed 
      Px4 
Tentative 
Levels: Tentative Confirmed Rejected
```


```
print(Bor.prdx.m.imp.selected)
```


```
[1] TRUE TRUE TRUE TRUE TRUE TRUE TRUE
```


```
rf.prdx.list <- list()
for (i in 1:10) {
  randomForest(x=X.prdx.x.m[,Bor.prdx.m.selected],y=as.factor(as.character(Y.prdx)),importance = T) -> rf.prdx.list[[i]]
  #print(rf.prdx.list[[i]])
}
rf.prdx.avg.conf  <- rf.prdx.list[[1]]$confusion
for (i in 2:10) rf.prdx.avg.conf <- rf.prdx.avg.conf + rf.prdx.list[[i]]$confusion
rf.prdx.avg.conf <- rf.prdx.avg.conf/10
print(rf.prdx.avg.conf)
```


```
     Ctrl IgAN  LN  MN class.error
Ctrl  0.0  0.0 3.0 4.0   1.0000000
IgAN  1.0  9.8 1.0 4.2   0.3875000
LN    0.1  1.0 6.9 4.0   0.4250000
MN    2.8  3.9 4.3 7.0   0.6111111
```


```
rf.prdx.avg.oob  <- rf.prdx.list[[1]]$err.rate[500,1]
for (i in 2:10) rf.prdx.avg.oob <- rf.prdx.avg.oob + rf.prdx.list[[i]]$err.rate[500,1]
rf.prdx.avg.oob <- rf.prdx.avg.oob/10
print(rf.prdx.avg.oob)
```


```
      OOB 
0.5528302
```


```
rf.prdx.avg.imp  <- rf.prdx.list[[1]]$importance
for (i in 2:10) rf.prdx.avg.imp <- rf.prdx.avg.imp + rf.prdx.list[[i]]$importance
rf.prdx.avg.imp <- rf.prdx.avg.imp/10
print(rf.prdx.avg.imp[order(rf.prdx.avg.imp[,5],decreasing = T),1:5])
```


```
             Ctrl         IgAN           LN            MN
OPN  -0.005760000 1.131583e-01 -0.001549531 -0.0079518254
WBC   0.002746667 3.953437e-02  0.051256342 -0.0145082628
Px3  -0.017656667 9.068543e-05  0.055976429  0.0204282490
Hb   -0.010420000 7.591105e-02  0.037345397 -0.0373285420
eGFR  0.068183333 1.848285e-02 -0.008262944  0.0103232950
Px1   0.007470000 1.777880e-02 -0.003732294 -0.0002050938
Px4  -0.048990000 4.808730e-04  0.053642778 -0.0004673859
     MeanDecreaseAccuracy
OPN           0.028706517
WBC           0.016474627
Px3           0.016249679
Hb            0.014806674
eGFR          0.014644621
Px1           0.005220511
Px4           0.004409157
```


Despite very small number of objects it is possible to build non-random classifier, but quality of classification generally went down. The prediction for Control class is now much worse, on the other hand prediction of MN has improved significantly, prediction error is now about 50% instead of more than 80%.

OPN contributes mostly to recognition of IgAN, whereas PRDX3 contributes to prediction of MN. PRDX4 and PRDX2 are much weaker, just borderline relevant.

LS0tCnRpdGxlOiAiUiBOb3RlYm9vayIKb3V0cHV0OiBodG1sX25vdGVib29rCi0tLQoKCmBgYHtyfQpsaWJyYXJ5KCJvcGVueGxzeCIpCnJlYWQueGxzeCgiTm93eV9hbGwueGxzeCIsY29sTmFtZXMgPSBUKSAtPiBYCmxpYnJhcnkocmFuZG9tRm9yZXN0KQpsaWJyYXJ5KEJvcnV0YSkKWCRHcm91cCA8LSBhcy5mYWN0b3IoWCRHcm91cCkKWCRHZW5kZXIgPC0gYXMuZmFjdG9yKFgkR2VuZGVyKQpYJGVHRlJncm91cCA8LSBhcy5mYWN0b3IoWCRlR0ZSZ3JvdXApCmBgYAoKVHlwZSBvZiBjb25kaXRpb24gYXMgYSBkZWNpc2lvbiB2YXJpYWJsZQpGaXJzdCAgcGFydCAtIHdlIHRha2Ugb25seSB0aGVzZSB2YXJpYWJsZXMgd2l0aCBjb21wbGV0ZSBjb3ZlcmFnZSBvZiBhbGwgcGF0aWVudHMKVGhlbiB3ZSByZW1vdmUgcGF0aWVudCBJRCBhbmQgaWxuZXNzIHR5cGUgZnJvbSBkZXNjcmlwdG9ycy4gCkZpbmFsbHksIHdlIHRha2Ugb250byBhY2NvdW50IG9ubHkgdGhlIGF2ZXJhZ2VzIG9mIG1lYXN1cmVtZW50cyBmb3IgYWxsIHZhcmlhYmxlcywgYW5kIHJlbmFtZSB2YXJpYWJsZXMuIApgYGB7cn0KTWFzay5uYSA8LSB2ZWN0b3IoImxvZ2ljYWwiLGRpbShYKVsyXSkKZm9yIChpIGluIDE6ZGltKFgpWzJdKSBNYXNrLm5hW2ldIDwtIHN1bShpcy5uYShYWyxpXSkpPjAKWC5uZXcgPC0gWFssIU1hc2submFdClgubmV3IDwtIFgubmV3WywtMV0KWC5uZXcueCA8LSBYLm5ld1ssLTFdClgubmV3LnggPC0gWC5uZXcueFssLTJdCnByaW50KGRpbShYLm5ldy54KSkKcHJpbnQobmFtZXMoWC5uZXcueCkpCm5hbWVzKFgubmV3LngpIDwtIGMoIkduZHIiLCJCTUkiLCJDUiIsImVHRlIiLCJIYiIsIlBMVCIsIldCQyIsIk9QTiIpCnByaW50KG5hbWVzKFgubmV3LngpKQoKYGBgCk5vdyB3ZSBwZXJmb3JtIHRlc3RzIApGaXJzdCB3ZSBzaW1wbHkgdGVzdCB3aGljaCB2YXJpYWJsZXMgY29udHJpYnV0ZSBmb3IgcHJlZGljdGlvbiBvZiBpbG5lc3MgdHlwZS4gV2Ugc2V0IGEgc2VlZCB0byBhIGNvbnN0YW50IHZhbHVlIHRvIGdldCByZXN1bHRzIHRoYXQgYXJlIGV4YWNsdHkgcmVwcm9kdWNpYmxlLiAgCmBgYHtyfQpZLm5ldy40YyA8LSBYLm5ld1ssMV0KcmFuZG9tRm9yZXN0KHg9WC5uZXcueCx5PWFzLmZhY3Rvcihhcy5jaGFyYWN0ZXIoWS5uZXcuNGMpKSxpbXBvcnRhbmNlID0gVCkgLT4gcmYuNGMgCnByaW50KHJmLjRjKQpwcmludChyZi40YykKc2V0LnNlZWQoMjAyMikKQm9ydXRhKHg9WC5uZXcueCx5PWFzLmZhY3Rvcihhcy5jaGFyYWN0ZXIoWS5uZXcuNGMpKSxkb1RyYWNlID0gMSkgLT4gQm9yLjRjCnBsb3QoQm9yLjRjKQpCb3IuNGMkZmluYWxEZWNpc2lvbiAhPSAiUmVqZWN0ZWQiIC0+IEJvci40Yy5zZWxlY3RlZApwcmludChCb3IuNGMkZmluYWxEZWNpc2lvbikKcHJpbnQoQm9yLjRjLnNlbGVjdGVkKQpyZi40Yy5saXN0IDwtIGxpc3QoKQpmb3IgKGkgaW4gMToxMCkgewogIHJhbmRvbUZvcmVzdCh4PVgubmV3LnhbLEJvci40Yy5zZWxlY3RlZF0seT1hcy5mYWN0b3IoYXMuY2hhcmFjdGVyKFkubmV3LjRjKSksaW1wb3J0YW5jZSA9IFQpIC0+IHJmLjRjLmxpc3RbW2ldXQogICNwcmludChyZi40Yy5saXN0W1tpXV0pCn0KcmYuNGMuYXZnLmNvbmYgIDwtIHJmLjRjLmxpc3RbWzFdXSRjb25mdXNpb24KZm9yIChpIGluIDI6MTApIHJmLjRjLmF2Zy5jb25mIDwtIHJmLjRjLmF2Zy5jb25mICsgcmYuNGMubGlzdFtbaV1dJGNvbmZ1c2lvbgpyZi40Yy5hdmcuY29uZiA8LSByZi40Yy5hdmcuY29uZi8xMApwcmludChyZi40Yy5hdmcuY29uZikKcmYuNGMuYXZnLm9vYiAgPC0gcmYuNGMubGlzdFtbMV1dJGVyci5yYXRlWzUwMCwxXQpmb3IgKGkgaW4gMjoxMCkgcmYuNGMuYXZnLm9vYiA8LSByZi40Yy5hdmcub29iICsgcmYuNGMubGlzdFtbaV1dJGVyci5yYXRlWzUwMCwxXQpyZi40Yy5hdmcub29iIDwtIHJmLjRjLmF2Zy5vb2IvMTAKcHJpbnQocmYuNGMuYXZnLm9vYikKcmYuNGMuYXZnLmltcCAgPC0gcmYuNGMubGlzdFtbMV1dJGltcG9ydGFuY2UKZm9yIChpIGluIDI6MTApIHJmLjRjLmF2Zy5pbXAgPC0gcmYuNGMuYXZnLmltcCArIHJmLjRjLmxpc3RbW2ldXSRpbXBvcnRhbmNlCnJmLjRjLmF2Zy5pbXAgPC0gcmYuNGMuYXZnLmltcC8xMApwcmludChyZi40Yy5hdmcuaW1wW29yZGVyKHJmLjRjLmF2Zy5pbXBbLDVdLGRlY3JlYXNpbmcgPSBUKSwxOjVdKQpgYGAKClJhbmRvbWlzYXRpb24gZXhwZXJpbWVudAoKCmBgYHtyfQpyYW5kLnJmIDwtIGxpc3QoTlVMTCkKIApmb3IgKGkgaW4gMToxMCkgewogIFkucmFuZCA8LSBzYW1wbGUoWS5uZXcuNGMsODAscmVwbGFjZSA9IEYpCiAgcmFuZG9tRm9yZXN0KHg9WC5uZXcueFssQm9yLjRjLnNlbGVjdGVkXSx5PWFzLmZhY3Rvcihhcy5jaGFyYWN0ZXIoWS5yYW5kKSksaW1wb3J0YW5jZSA9IFQpIC0+IHJhbmQucmZbW2ldXQp9CnJmLnJhbmQuY29uZiAgPC0gcmFuZC5yZltbMV1dJGNvbmZ1c2lvbgpmb3IgKGkgaW4gMjoxMCkgcmYucmFuZC5jb25mIDwtIHJmLnJhbmQuY29uZiArIHJhbmQucmZbW2ldXSRjb25mdXNpb24KcmYucmFuZC5jb25mIDwtIHJmLnJhbmQuY29uZi8xMApwcmludChyZi5yYW5kLmNvbmYpCnJmLnJhbmQub29iICA8LSByYW5kLnJmW1sxXV0kZXJyLnJhdGVbNTAwLDFdCmZvciAoaSBpbiAyOjEwKSByZi5yYW5kLm9vYiA8LSByZi5yYW5kLm9vYiArIHJhbmQucmZbW2ldXSRlcnIucmF0ZVs1MDAsMV0KcmYucmFuZC5vb2IgPC0gcmYucmFuZC5vb2IvMTAKcHJpbnQocmYucmFuZC5vb2IpCnJmLnJhbmQuaW1wICA8LSByYW5kLnJmW1sxXV0kaW1wb3J0YW5jZQpmb3IgKGkgaW4gMjoxMCkgcmYucmFuZC5pbXAgPC0gcmYucmFuZC5pbXAgKyByYW5kLnJmW1tpXV0kaW1wb3J0YW5jZQpyZi5yYW5kLmltcCA8LSByZi5yYW5kLmltcC8xMApwcmludChyZi5yYW5kLmltcFtvcmRlcihyZi5yYW5kLmltcFssNV0sZGVjcmVhc2luZyA9IFQpLDE6NV0pCgoKYGBgCgpXZSBjYW4gc2VlLCB0aGF0IHdpdGggcmFuZG9taXNlZCB2ZXJzaW9uIG9mIHZhcmlhYmxlcyB3ZSBnZXQgcmFuZG9tIGNsYXNzaWZpZXIgd2l0aCBubyByZWxldmFudCB2YXJpYWJsZXMuIAoKCk5vdyB3ZSBjaGVjayB3aGV0aGVyIHdlIGNhbiBkaXNjZXJuIElnQU4gZnJvbSBvdGhlciBjbGFzc2VzIApgYGB7cn0KWS5uZXcuaWdhbiA8LSBYLm5ld1ssMV0gPT0gIklnQU4iCnNldC5zZWVkKDIwMjIpCnJhbmRvbUZvcmVzdCh4PVgubmV3LngseT1hcy5mYWN0b3IoWS5uZXcuaWdhbiksaW1wb3J0YW5jZSA9IFQpIC0+IHJmLmlnYW4gCnByaW50KHJmLmlnYW4pCnByaW50KHJmLmlnYW4pCkJvcnV0YSh4PVgubmV3LngseT1hcy5mYWN0b3IoYXMuY2hhcmFjdGVyKFkubmV3LmlnYW4pKSxkb1RyYWNlID0gMSkgLT4gQm9yLmlnYW4KcGxvdChCb3IuaWdhbikKQm9yLmlnYW4kZmluYWxEZWNpc2lvbiAhPSAiUmVqZWN0ZWQiIC0+IEJvci5pZ2FuLnNlbGVjdGVkCnByaW50KEJvci5pZ2FuJGZpbmFsRGVjaXNpb24pCnByaW50KEJvci5pZ2FuLnNlbGVjdGVkKQpyZi5pZ2FuLmxpc3QgPC0gbGlzdCgpCmZvciAoaSBpbiAxOjEwKSB7CiAgcmFuZG9tRm9yZXN0KHg9WC5uZXcueFssQm9yLmlnYW4uc2VsZWN0ZWRdLHk9YXMuZmFjdG9yKGFzLmNoYXJhY3RlcihZLm5ldy5pZ2FuKSksaW1wb3J0YW5jZSA9IFQpIC0+IHJmLmlnYW4ubGlzdFtbaV1dCiAgI3ByaW50KHJmLmlnYW4ubGlzdFtbaV1dKQp9CnJmLmlnYW4uYXZnLmNvbmYgIDwtIHJmLmlnYW4ubGlzdFtbMV1dJGNvbmZ1c2lvbgpmb3IgKGkgaW4gMjoxMCkgcmYuaWdhbi5hdmcuY29uZiA8LSByZi5pZ2FuLmF2Zy5jb25mICsgcmYuaWdhbi5saXN0W1tpXV0kY29uZnVzaW9uCnJmLmlnYW4uYXZnLmNvbmYgPC0gcmYuaWdhbi5hdmcuY29uZi8xMApwcmludChyZi5pZ2FuLmF2Zy5jb25mKQpyZi5pZ2FuLmF2Zy5vb2IgIDwtIHJmLmlnYW4ubGlzdFtbMV1dJGVyci5yYXRlWzUwMCwxXQpmb3IgKGkgaW4gMjoxMCkgcmYuaWdhbi5hdmcub29iIDwtIHJmLmlnYW4uYXZnLm9vYiArIHJmLmlnYW4ubGlzdFtbaV1dJGVyci5yYXRlWzUwMCwxXQpyZi5pZ2FuLmF2Zy5vb2IgPC0gcmYuaWdhbi5hdmcub29iLzEwCnByaW50KHJmLmlnYW4uYXZnLm9vYikKcmYuaWdhbi5hdmcuaW1wICA8LSByZi5pZ2FuLmxpc3RbWzFdXSRpbXBvcnRhbmNlCmZvciAoaSBpbiAyOjEwKSByZi5pZ2FuLmF2Zy5pbXAgPC0gcmYuaWdhbi5hdmcuaW1wICsgcmYuaWdhbi5saXN0W1tpXV0kaW1wb3J0YW5jZQpyZi5pZ2FuLmF2Zy5pbXAgPC0gcmYuaWdhbi5hdmcuaW1wLzEwCnByaW50KHJmLmlnYW4uYXZnLmltcFtvcmRlcihyZi5pZ2FuLmF2Zy5pbXBbLDNdLGRlY3JlYXNpbmcgPSBUKSwxOjNdKQpgYGAKClllcywgd2UgY2FuLiBGaXZlIHZhcmlhYmxlcyBjb250cmlidXRlLiBCeSBmYXIgbW9zdCByZWxldmFudCB2YXJpYWJsZSBpcyBPUE4uCgpOb3cgd2Ugd2lsbCByZXBlYXQgdGhlIGFuYWx5c2VzIHdpdGggZGF0YSBzZXQgbGltaXRlZCB0byBwYXRpZW50cyB3aG8gaGF2ZSBQUkRYeCBtZWFzdXJlbWVudHMsIGFuZCBhbGwgdmFyaWFibGVzIHRoYXQgd2VyZSByZWNvcmRlZCBmb3IgdGhlc2UgcGF0aWVudHMgd2lsbCBiZSBpbmNsdWRlZC4gCgpgYGB7cn0KTWFzay5wcmR4IDwtICFpcy5uYShYJFBSRFgxKQpYLnByZHggPC0gWFtNYXNrLnByZHgsXQpzZXQuc2VlZCgyMDIyKQpNYXNrLm5hIDwtIHZlY3RvcigibG9naWNhbCIsZGltKFgucHJkeClbMl0pCmZvciAoaSBpbiAxOmRpbShYKVsyXSkgTWFzay5uYVtpXSA8LSBzdW0oaXMubmEoWC5wcmR4WyxpXSkpPjAKClgucHJkeCA8LSBYLnByZHhbLCFNYXNrLm5hXQpYLnByZHgueCA8LSBYLnByZHhbLGMoLTEsLTIsLTQsLTUsLTgsLTksLTE3KV0KIyB3ZSByZW1vdmUgYWxsIG5vbiBtZWFuIG1lYXN1cmVtZW50cyAKWC5wcmR4LngubSA8LSBYLnByZHgueFssYygtMiwtMyldCm5hbWVzKFgucHJkeC54Lm0pICA8LSBjKCJHbmRyIiwiQk1JIiwiQ1IiLCJlR0ZSIiwiSGIiLCJQTFQiLCJXQkMiLCJPUE4iLCJQeDEiLCJQeDIiLCJQeDMiLCJQeDQiLCJQeDUiKQoKWS5wcmR4IDwtIFgucHJkeCRHcm91cAoKcmFuZG9tRm9yZXN0KHg9WC5wcmR4LngseT1hcy5mYWN0b3IoWS5wcmR4KSxpbXBvcnRhbmNlID0gVCkgLT4gcmYucHJkeCAKcHJpbnQocmYucHJkeCkKcmFuZG9tRm9yZXN0KHg9WC5wcmR4LngubSx5PWFzLmZhY3RvcihZLnByZHgpLGltcG9ydGFuY2UgPSBUKSAtPiByZi5wcmR4Lm0KcHJpbnQocmYucHJkeC5tKQpCb3J1dGEoeD1YLnByZHgueCx5PWFzLmZhY3Rvcihhcy5jaGFyYWN0ZXIoWS5wcmR4KSksZG9UcmFjZSA9IDEpIC0+IEJvci5wcmR4CnBsb3QoQm9yLnByZHgpCkJvci5wcmR4JGZpbmFsRGVjaXNpb24gIT0gIlJlamVjdGVkIiAtPiBCb3IucHJkeC5zZWxlY3RlZApwcmludChCb3IucHJkeCRmaW5hbERlY2lzaW9uKQpwcmludChCb3IucHJkeC5zZWxlY3RlZCkKIwojIFNtYWxsZXIgZGF0YSBzZXQKIwpCb3J1dGEoeD1YLnByZHgueC5tLHk9YXMuZmFjdG9yKGFzLmNoYXJhY3RlcihZLnByZHgpKSxkb1RyYWNlID0gMSkgLT4gQm9yLnByZHgubQpwbG90KEJvci5wcmR4Lm0pCkJvci5wcmR4Lm0kZmluYWxEZWNpc2lvbiAhPSAiUmVqZWN0ZWQiIC0+IEJvci5wcmR4Lm0uc2VsZWN0ZWQKcHJpbnQoQm9yLnByZHgubSRmaW5hbERlY2lzaW9uKQpwcmludChCb3IucHJkeC5tLnNlbGVjdGVkKQoKWC5wcmR4LngubS5pbXAgPC0gWC5wcmR4LngubVssQm9yLnByZHgubS5zZWxlY3RlZF0KQm9ydXRhKHg9WC5wcmR4LngubS5pbXAseT1hcy5mYWN0b3IoYXMuY2hhcmFjdGVyKFkucHJkeCkpLGRvVHJhY2UgPSAxKSAtPiBCb3IucHJkeC5tLmltcApwbG90KEJvci5wcmR4Lm0uaW1wKQpCb3IucHJkeC5tLmltcCRmaW5hbERlY2lzaW9uICE9ICJSZWplY3RlZCIgLT4gQm9yLnByZHgubS5pbXAuc2VsZWN0ZWQKcHJpbnQoQm9yLnByZHgubS5pbXAkZmluYWxEZWNpc2lvbikKcHJpbnQoQm9yLnByZHgubS5pbXAuc2VsZWN0ZWQpCgpyZi5wcmR4Lmxpc3QgPC0gbGlzdCgpCmZvciAoaSBpbiAxOjEwKSB7CiAgcmFuZG9tRm9yZXN0KHg9WC5wcmR4LngubVssQm9yLnByZHgubS5zZWxlY3RlZF0seT1hcy5mYWN0b3IoYXMuY2hhcmFjdGVyKFkucHJkeCkpLGltcG9ydGFuY2UgPSBUKSAtPiByZi5wcmR4Lmxpc3RbW2ldXQogICNwcmludChyZi5wcmR4Lmxpc3RbW2ldXSkKfQpyZi5wcmR4LmF2Zy5jb25mICA8LSByZi5wcmR4Lmxpc3RbWzFdXSRjb25mdXNpb24KZm9yIChpIGluIDI6MTApIHJmLnByZHguYXZnLmNvbmYgPC0gcmYucHJkeC5hdmcuY29uZiArIHJmLnByZHgubGlzdFtbaV1dJGNvbmZ1c2lvbgpyZi5wcmR4LmF2Zy5jb25mIDwtIHJmLnByZHguYXZnLmNvbmYvMTAKcHJpbnQocmYucHJkeC5hdmcuY29uZikKcmYucHJkeC5hdmcub29iICA8LSByZi5wcmR4Lmxpc3RbWzFdXSRlcnIucmF0ZVs1MDAsMV0KZm9yIChpIGluIDI6MTApIHJmLnByZHguYXZnLm9vYiA8LSByZi5wcmR4LmF2Zy5vb2IgKyByZi5wcmR4Lmxpc3RbW2ldXSRlcnIucmF0ZVs1MDAsMV0KcmYucHJkeC5hdmcub29iIDwtIHJmLnByZHguYXZnLm9vYi8xMApwcmludChyZi5wcmR4LmF2Zy5vb2IpCnJmLnByZHguYXZnLmltcCAgPC0gcmYucHJkeC5saXN0W1sxXV0kaW1wb3J0YW5jZQpmb3IgKGkgaW4gMjoxMCkgcmYucHJkeC5hdmcuaW1wIDwtIHJmLnByZHguYXZnLmltcCArIHJmLnByZHgubGlzdFtbaV1dJGltcG9ydGFuY2UKcmYucHJkeC5hdmcuaW1wIDwtIHJmLnByZHguYXZnLmltcC8xMApwcmludChyZi5wcmR4LmF2Zy5pbXBbb3JkZXIocmYucHJkeC5hdmcuaW1wWyw1XSxkZWNyZWFzaW5nID0gVCksMTo1XSkKCmBgYAoKRGVzcGl0ZSB2ZXJ5IHNtYWxsIG51bWJlciBvZiBvYmplY3RzIGl0IGlzIHBvc3NpYmxlIHRvIGJ1aWxkIG5vbi1yYW5kb20gY2xhc3NpZmllciwgYnV0IHF1YWxpdHkgb2YgY2xhc3NpZmljYXRpb24gZ2VuZXJhbGx5IHdlbnQgZG93bi4gVGhlIHByZWRpY3Rpb24gZm9yIENvbnRyb2wgY2xhc3MgaXMgbm93IG11Y2ggd29yc2UsIG9uIHRoZSBvdGhlciBoYW5kIHByZWRpY3Rpb24gb2YgTU4gaGFzIGltcHJvdmVkIHNpZ25pZmljYW50bHksIHByZWRpY3Rpb24gZXJyb3IgaXMgbm93IGFib3V0IDUwJSBpbnN0ZWFkIG9mIG1vcmUgdGhhbiA4MCUuIAoKT1BOIGNvbnRyaWJ1dGVzIG1vc3RseSB0byByZWNvZ25pdGlvbiBvZiBJZ0FOLCB3aGVyZWFzIFBSRFgzIGNvbnRyaWJ1dGVzIHRvIHByZWRpY3Rpb24gb2YgTU4uIApQUkRYNCBhbmQgUFJEWDIgYXJlIG11Y2ggd2Vha2VyLCBqdXN0IGJvcmRlcmxpbmUgcmVsZXZhbnQuIAoKCg==
