# Supplementary material for: Osteopontin—A Potential Biomarker for IgA Nephropathy: Machine Learning Application
Source: Biomedicines. 2022 Mar 22;10(4):734. doi: 10.3390/biomedicines10040734 (PMC9025015; doi:10.3390/biomedicines10040734)
Supplement: Supplementary file 1 [file biomedicines-10-00734-s001.zip › Table S1.pdf]

Supplementary Table S1. Correlations between OPN levels (1st sampling point) and clinical parameters using Spearmann's correlation analysis.

Parameter	IgAN (n=29)			LN (n=18)			MN (n=20)			Control (n=13)		
	R	R^2	p	R	R^2	p	R	R^2	p	R	R^2	p
Single measurement (1st sampling point)												
**Age (years)**	0.066	0.004	0.734	-0.249	0.062	0.319	-0.334	0.111	0.150	-0.063	0.004	0.837
**BMI (kg/m²)**	-0.024	0.001	0.903	**-0.488**	**0.238**	**0.040**	0.386	0.149	0.092	0.093	0.009	0.762
**WBC (G/L)**	-0.024	0.001	0.903	-0.218	0.047	0.385	0.269	0.072	0.252	0.542	0.294	0.056
**HGB (g/dL)**	0.178	0.032	0.355	0.279	0.078	0.262	-0.114	0.013	0.633	0.320	0.102	0.287
**PLT (G/L)**	0.033	0.001	0.864	-0.168	0.028	0.505	0.023	0.001	0.925	0.165	0.027	0.590
**Serum creatinine (mg/dL)**	-0.240	0.058	0.209	-0.417	0.174	0.085	-0.232	0.054	0.326	0.044	0.002	0.887
**eGFR (mL/min×1.73m²)**	0.181	0.033	0.348	0.406	0.164	0.095	0.284	0.081	0.225	0.253	0.064	0.405
**Proteinuria (g/24h)**	-0.074	0.006	0.707	-0.257	0.066	0.375	0.172	0.030	0.494	n.a.	n.a.	n.a.
Long-term follow-up (mean values of all observations)												
**BMI (kg/m²)**	0.006	4×10^{-5}	0.974	**-0.540**	**0.291**	**0.021**	0.218	0.048	0.356	0.093	0.009	0.762
**WBC (G/L)**	-0.082	0.007	0.673	0.125	0.016	0.622	0.101	0.010	0.673	0.341	0.116	0.255
**HGB (g/dL)**	0.277	0.077	0.145	0.099	0.010	0.696	0.014	$1.8\text{x}10^{-4}$	0.955	0.286	0.082	0.344
**PLT (G/L)**	-0.100	0.010	0.608	0.135	0.018	0.593	-0.033	0.001	0.890	-0.055	0.003	0.859
**Serum creatinine (mg/dL)**	-0.240	0.058	0.209	-0.364	0.133	0.137	-0.024	0.001	0.920	0.074	0.006	0.809
**eGFR (mL/min×1.73m²)**	0.211	0.044	0.272	0.313	0.098	0.206	0.110	0.012	0.645	-0.016	3×10^{-4}	0.957
**Proteinuria (g/24h)**	-0.153	0.023	0.428	0.042	0.002	0.868	0.277	0.077	0.238	n.a.	n.a.	n.a.
