# Supplementary material for: Osteopontin—A Potential Biomarker for IgA Nephropathy: Machine Learning Application
Source: Biomedicines. 2022 Mar 22;10(4):734. doi: 10.3390/biomedicines10040734 (PMC9025015; doi:10.3390/biomedicines10040734)
Supplement: Supplementary file 1 [file biomedicines-10-00734-s001.zip › Tables S2-S4.pdf]

We obtained biopsy results assessing disease severity in light microscopy of 23/29 IgAN (Table S4), 20/20 MN (Table S5) and 13/18 LN patients (Table S6). All 23 IgAN patients were evaluated using Hass classification and Oxford classification. The full description of other patients' biopsies was not available, as they were performed in other centers.

Supplementary Table S2. Combined Hass and Oxford classifications of IgAN.

Oxford Classification, (n)	*Hass Classification, (n)*				
	**I**	**II**	**III**	**IV**	**V**
	**n=0**	**n=5**	**n=10**	**n=6**	**n=2**
M					
**0**	n.a.	5	9	1	1
**1**	n.a.	0	1	5	1
E					
**0**	n.a.	5	5	4	1
**1**	n.a.	0	5	2	1
S					
**0**	n.a.	0	2	0	0
**1**	n.a.	5	8	6	2
T					
**0**	n.a.	4	9	5	0
**1**	n.a.	1	1	1	2
C					
**0**	n.a.	5	7	3	1
**1**	n.a.	0	3	3	1

n = number of patients

Supplementary Table S3. MN stages (light microscopy).

Stages,	*(n)*	*Serum PLA2R, (n)*	
**I**	5	positive (7)	negative (13)
**I/II**	3		
**II**	5		
**II/III**	5		
**III**	2		
**IV**	0		

n = number of patients

Supplementary Table S4. LN stages (light microscopy).

Stages,	*(n)*
**I**	0
**II**	1
**III**	4
**III/V**	1
**IV**	5
**IV/V**	1
**V**	1
**VI**	0

n = number of patients
